# Direct Measurement of Protein Pair Interaction Potential

**DOI:** 10.1021/acsnano.5c19213

**Published:** 2026-03-06

**Authors:** Ekaterina Poliukhina, Quy Ong, Davide Demurtas, Emiko Uchikawa, Notash Shafie, Francesco Stellacci

**Affiliations:** † Laboratory of Supramolecular Nanomaterials and Interfaces, Ecole Polytechnique Fédérale de Lausanne (EPFL), Lausanne 1015, Switzerland; ‡ Interdisciplinary Centre for Electron Microscopy (CIME), Swiss Federal Institute of Technology Lausanne (EPFL), Lausanne 1015, Switzerland; § Dubochet Center for Imaging (DCI), 27218 Ecole Polytechnique Fédérale de Lausanne (EPFL) and University of Lausanne, Lausanne 1015, Switzerland; ∥ Institute of Bioengineering, École Polytechnique Fédérale de Lausanne (EPFL), Station 12, Lausanne 1015, Switzerland; ⊥ Global Health Institute, École Polytechnique Fédérale de Lausanne (EPFL), Station 12, Lausanne 1015, Switzerland

**Keywords:** protein−protein interactions, cryogenic electron tomography, potential of mean force, pair interaction potential, globular protein, Kirkwood−Buff integral

## Abstract

A direct and unambiguous method for obtaining the pair interaction potential (PIP) of proteins does not currently exist. All existing approaches require solving an inverse problem, which always allows for alternative solutions. Here, we report a straightforward method for obtaining the PIP directly from experimentally determined three-dimensional spatial distributions of proteins. The approach is based on improvements to a recently developed method for determining the potential of mean force for nanoparticles using cryogenic electron tomography (cryo-ET). For the protein PIP, we find good agreement between the structure factor computed from cryo-ET positions and that obtained from small-angle X-ray scattering of protein solutions. We apply a novel subvolume method to compute Kirkwood–Buff integrals and show that the second virial coefficients calculated from the cryo-ET tomograms closely match those obtained experimentally from analytical ultracentrifugation. Both results validate our approach for deriving the PIP and indicate that the vitrified state matches the solution state. The generality of our validated approach is demonstrated for several small proteins with different structures and molecular weights, and under various experimental conditions, including changes in salt concentration, temperature, and pH. Our method does not require assumptions about protein shape or the analytical form of the interaction potential.

## Graphical abstract

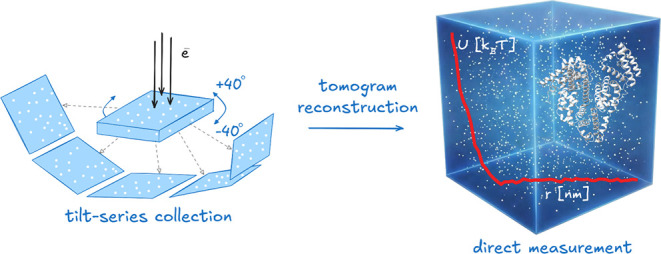

## Introduction

Protein–protein interactions (**PPIs**) play a crucial role in determining protein functions and properties, including aggregation, agglomeration, viscosity, and phase separation.^1–6^ Understanding the principles that govern PPIs is therefore essential for deciphering biological processes and for advancing the fields of pharmacology and protein engineering.^7–10^ A key quantity describing PPIs is the pair interaction potential (**PIP**, *U*(*r*)) which, by definition, is the reversible work required to bring two proteins from infinite separation to a centroid-to-centroid distance *r*.^11^ At finite protein concentration *c*, the corresponding quantity is the potential of mean force (**PMF**, *W*(*r*,*c*)), which includes many-body contributions from surrounding proteins and is related to the PIP by *W*(*r*,*c*) = *U*(*r*) + Δ*W*(*r*,*c*), with Δ*W*(*r*,*c*) → 0 as *c* → 0; thus, the concentration-dependent PMF converges to the concentration-independent PIP in the infinite-dilution limit.^11^ Because the PIP provides the foundation for predicting protein thermodynamic properties and phase behaviors,^12^ its direct experimental determination is highly desirable.

Small-angle scattering (e.g., small-angle X-ray scattering, **SAXS**; or small-angle neutron scattering, **SANS**) is, so far, the only technique capable of providing a PIP for proteins by solving the inverse problem from their structure factor.^13–16^ However, this is possible only under the condition that both an interaction model and a closure relation are assumed *a priori*.^17^ As an indirect method, it cannot yield a unique PIP, and the results are often open to multiple interpretations.^18^ Despite the significance of the PIP, a direct method for measuring it has not yet been available.

Recently, we reported a method to obtain the PMF of generic colloidal nanoparticles using cryogenic electron tomography (**cryo-ET**).^19^ This method involves rapidly freezing a dispersion of nanoparticles to preserve their native state. While in a frozen state, the sample is imaged by transmission electron microscopy (**TEM**) at multiple tilt angles to obtain a tilt series. After alignment of the tilt series and tomogram reconstruction, the resulting three-dimensional (**3D**) image can be processed to accurately determine the centroid positions of all nanoparticles. From these 3D positions, the radial distribution function (**RDF**, *g*(*r*,*c*)) can be computed, which in turn yields the **PMF** of the nanoparticles using the well-known reversible work theorem:

$$W\left(r,c\right)=-k_{B}T\ln\left[g\left(r,c\right)\right]$$

where *k*_B_ is the Boltzmann constant and *T* is the temperature.^11^

The method is general and applicable to any nanocolloid, enabling the investigation of nanoparticle interactions in realistic environments.^19–23^ We demonstrated and validated this approach using gold nanoparticles (**AuNPs**) with an average diameter below 5 nm under various conditions. However, applying this method to proteins proved to be nontrivial. Several technical challenges arise that are either absent or easily addressed in the case of AuNPs (see Supporting Information, Tables SI1 and SI2). Notably, proteins present additional difficulties due to their low contrast,^24,25^ high surface activity,^26,27^ and tendency to aggregate^28,29^ —factors that require significant methodological developments beyond our previous work.

Here, we report the advancement of our cryo-ET method for application to proteins, particularly small proteins. We successfully reconstructed the 3D distributions of proteins in their vitrified native states, obtained their spatial 3D positions, and directly calculated the corresponding PMFs. We demonstrate its use across several globular proteins. Our approach is straightforward, model-free, and broadly applicable.

Over the concentration range explored here, the measured PMFs do not exhibit a clear dependence on protein concentration within experimental uncertainty, indicating negligible many-body corrections, Δ*W*(*r*,*c*) → 0; consequently, the obtained PMFs serve as protein effective PIPs. The ability to directly determine the PIP enables straightforward systematic investigations of the effects of salt concentration, pH, temperature, and the presence of small molecules such as the amino acid proline.

## Results

### PIP Measurement

Figure 1a illustrates the cryo-ET approach used to measure protein interaction potentials. This method follows our previously published procedure^19^ with several modifications for proteins, which are weak-contrast objects and highly active at the air–water interface (**AWI**) (see Methods and Table SI1 in the Supporting Information for details). Prior to microscopy experiments, proteins are purified by fast protein liquid chromatography (**FPLC**) to remove preexisting, covalently bound oligomers (e.g., dimers, trimers, and other irreversible high-molecular-weight species), thus allowing us to start from a well-defined monomeric population. This step does not remove the transient near-contact associations among monomers that define the measured PIP under the experimental conditions. Indeed, upon dilution of the unpurified sample the ratio between monomers and oligomers does not change, indicating preexisting irreversible aggregates, while after purification oligomers do not reform covalently even at high concentration after extended storage time (see Figure SI2, Table SI3).

**Figure 1:**
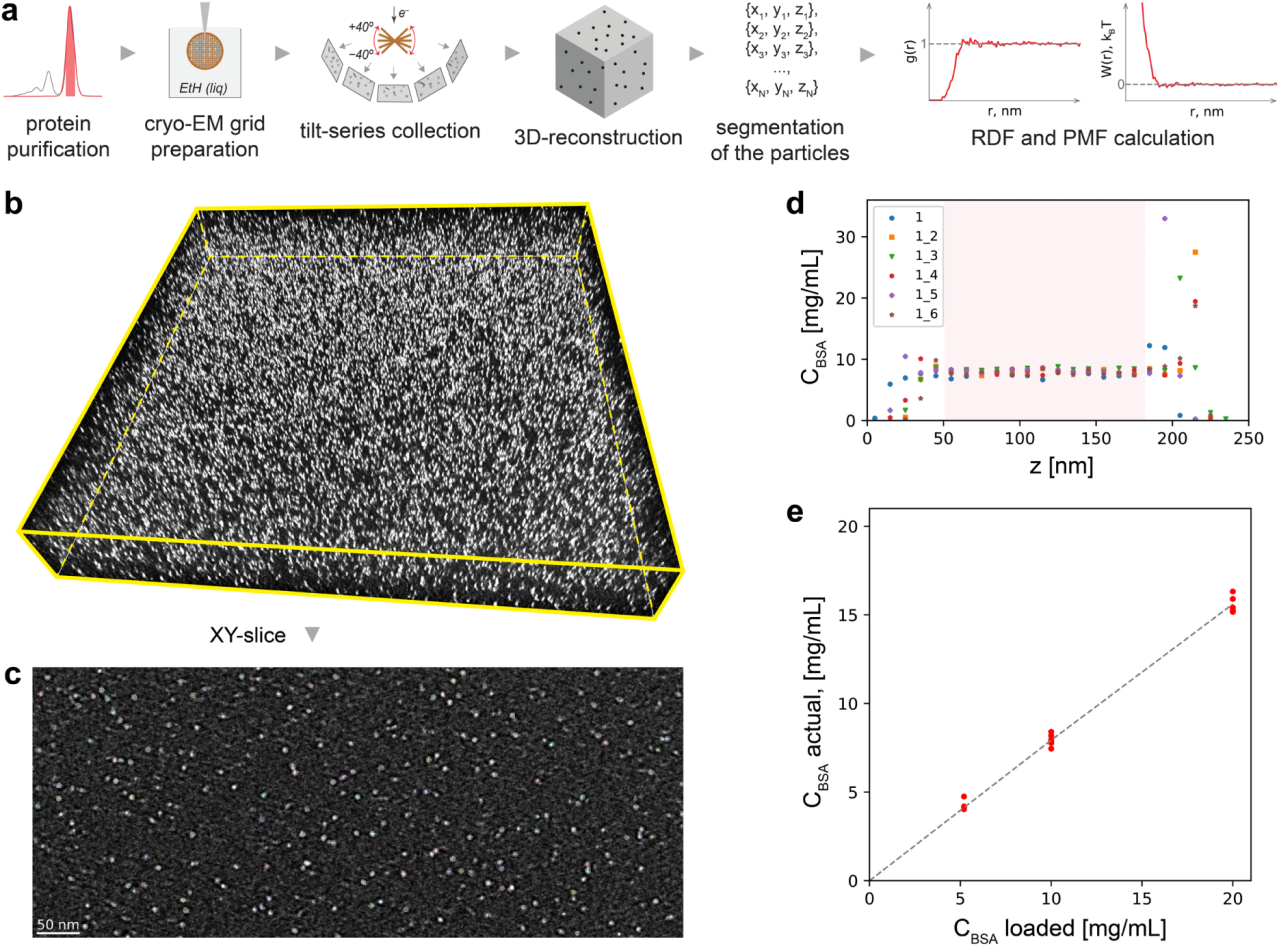
Cryo-ET method for the direct measurement of the PIP of proteins: (a) scheme of the experimental workflow; (b) a tomogram example for ∼10 mg/mL BSA in phosphate buffer; and (c) a 9 nm thick XY-slice with segmented protein particles in white; (d) dependence of the actual measured concentration of BSA inside the tomogram as a function of the height of the tomogram; the highlighted region indicates the height range used for RDF calculation; see Figure SI1 and Table SI2 for tilt correction and cropping of the tomogram. The legend indicates 6 different tomograms used in this plot. (e) Correlation between the actual measured concentration of BSA in the bulk of the tomogram and the concentration of the sample measured by spectrophotometry.

Grids are then prepared using a Chameleon instrument,^30^ which enables rapid, blot-free vitrification of the protein sample with controllable sample thickness. This approach substantially reduces undesired protein adsorption at the AWI and minimizes protein denaturation.

During imaging by TEM at liquid-nitrogen temperature, dose-symmetric tilt series are collected using high negative defocus values to enhance contrast, as our goal is to extract only the centroids of protein masses. The tilt series are subsequently aligned and reconstructed into 3D images, followed by particle segmentation. The extracted 3D positions of the protein centroids are then used to numerically calculate the RDF, which is finally converted into the PMF (see Table SI4).

Figure 1b shows a representative tomogram of BSA, a common globular protein, at a concentration of ∼10 mg/mL in 76 mM phosphate buffer. A 9 nm thick XY slice of this tomogram is presented in Figure 1c, clearly showing the detected particles. In both figures, the contrast is inverted so that protein particles appear as white ellipsoids, well-known as the missing wedge effect. It can be seen that inside the tomogram, the protein concentration remains consistent along its height for all positions analyzed (Figure 1d), indicating the absence of a concentration gradient. As expected, the protein concentration measured from the tomogram is lower than that of the original solution, primarily due to partial protein adsorption at the AWI. When 5.2 mg/mL, 10 mg/mL, and 20 mg/mL BSA solutions are used, the concentrations measured directly from their tomograms are approximately 4 mg/mL, 8 mg/mL, and 16 mg/mL, respectively (Figure 1e). Therefore, the ratio of loaded concentrations is also found to be constant, most likely because the small size and high diffusion coefficients of proteins, which promote a uniform distribution.

The averaged RDF and PMF curves for 4 mg/mL (black), 8 mg/mL (blue), and 16 mg/mL (red) BSA solutions were calculated and are shown in Figure 2a and b, respectively. The PMF curves of BSA are consistent with those expected for charge-stabilized soft spheres. We found that the PMF varies negligibly with concentration (Figure SI3). The same behavior is observed for many other proteins, see the section “PIP of Many Other Small Proteins” below. Thus, the measured PMFs essentially represent the effective protein pair interaction potential, PIP, or *U*(*r*).

**Figure 2:**
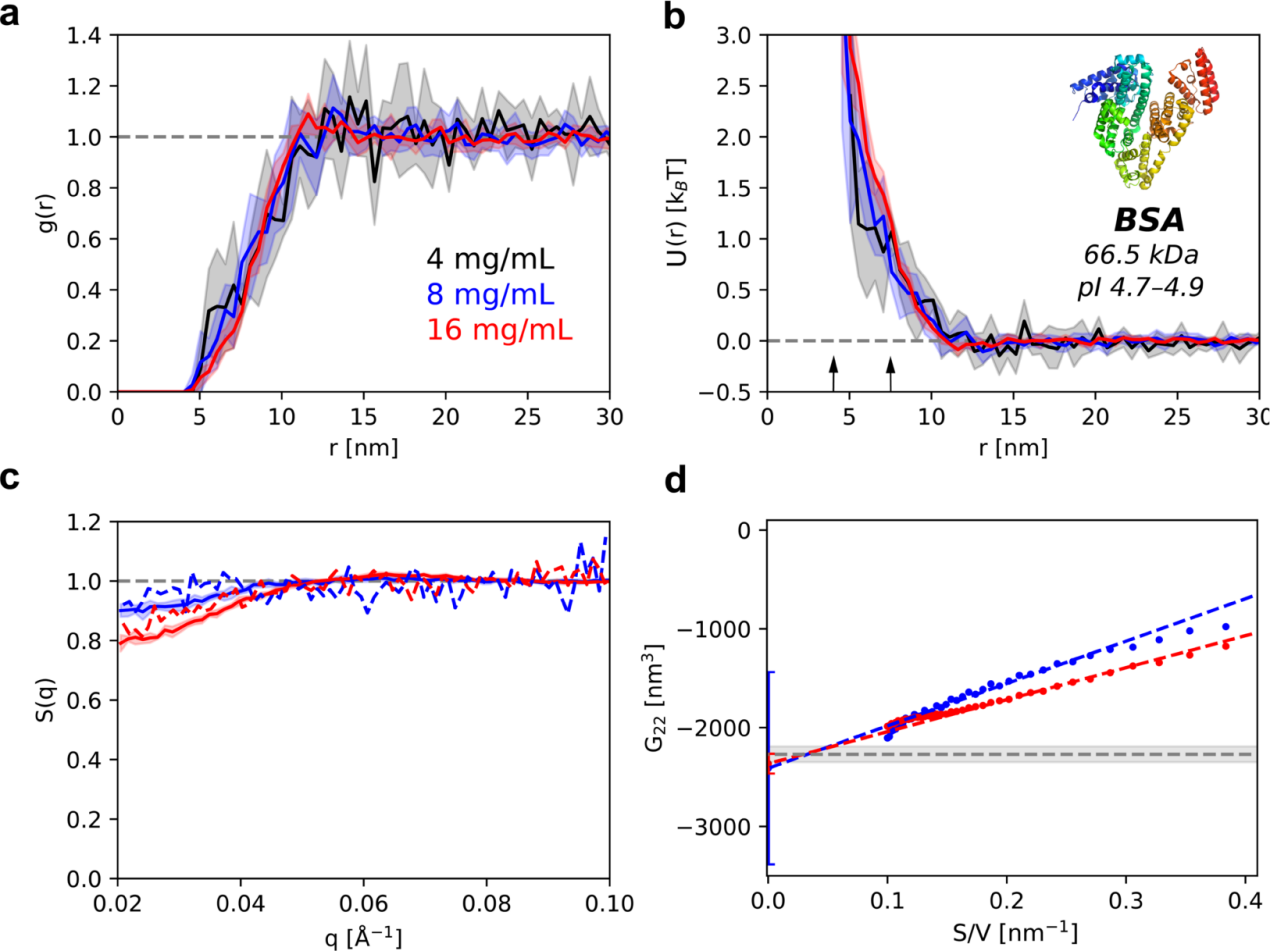
Pair interaction potential of BSA and validation of the results: (a, b) plots showing the dependence of the RDF and PMF, correspondingly, on centroid-to-centroid distance; the arrows indicate the smallest and largest dimensions of BSA as determined from its PDB structure; (c) comparison between the structure factor calculated from cryo-ET particle spatial positions (solid) and that measured by the SAXS method (dashed); (d) KBI calculated by sub-box Schnell’s approach^31–33^ (see Methods and Figure SI6); gray line shows the KBI recalculated from the second virial coefficient measured by AUC-SE. The sample at 4 mg/mL was not used for the calculation of the KBI because at that concentration there are too few particles in the box. Measurements correspond to BSA samples at 4 mg/mL (black), 8 mg/mL (blue), and 16 mg/mL (red) in 76 mM phosphate buffer at pH 7.2 and 20 °C. Shaded areas indicate the standard deviation across replicates.

It is necessary to validate the PIP results. Validation requires demonstrating that the measurements are conducted under equilibrium conditions and that the protein spatial distribution remains unchanged during the freezing process. To this end, we applied two analytical techniques orthogonal to cryo-ET—SAXS and AUC-SE—to the same protein solutions used in cryo-ET. The comparison between the structure factors *S*(*q*) of BSA, measured experimentally by SAXS (Figure SI4) and those calculated from cryo-ET positions, shows good agreement between the two methods (Figure 2c). The cross-validation technique used to measure the second virial coefficient *B*_22_ experimentally was AUC-SE (Figure SI5). We obtained the Kirkwood–Buff integral (**KBI**, or $G_{22}^{\infty }$ ), which is equivalent to *B*_22_, from cryo-ET by applying a novel sub-box method^31–33^ to the protein spatial positions (see Methods). The values of $G_{22}^{\infty }$ obtained for BSA in the dilute regime match the *B*_22_ obtained from the AUC-SE (Figure 2d). These two values are also comparable to the *B*_22_ derived from the concentration dependence of *S*(0) (Table 1), also directly from cryo-ET (Figure SI7). The cross-validation techniques used here confirm the reliability of our PIP measurements and indicate that the vitrified state represents a “frozen” solution equilibrium state.

**Table 1:** KBI Values in [nm^3^], Calculated from the Cryo-ET Spatial Distribution of Protein and Measured by AUC-SE Technique, Respectively

	Cryo-ET		
Concentration of BSA [mg/mL]	Sub-box method	Slope of *S*(0,ρ)	AUC-SE
8	–2411 ± 973	–2199 ± 212	–2260 ± 80
16	–2365 ± 100		

### PIPs of BSA in Various Conditions

To show the versatility of our method, we applied our validated cryo-ET method to BSA under various experimental conditions. BSA is often studied as a model protein, typically approximated as a soft spherical particle whose colloidal stability arises from its charge.^13–16^ This electrostatic repulsion can be modulated by temperature, the ionic strength of the medium, or by the pH of the medium which alters the protonation state and thus the net charge of BSA. We investigated how the PIP of BSA responds to changes in these three parameters.

Comparing conditions of 10 mM and 975 mM of added NaCl, we observe that increasing ionic strength reduces the interaction decay length from 13 to 10 nm and partially screens the repulsion by ∼0.5 *k*_B_ *T* (Figure 3a), as expected for the effect of salt-induced reduction of electrostatic repulsion.^34^ Despite the use of a high salt concentration, the interactions remain repulsive, indicating that the contribution to the stability of BSA in solution is more than simple electrostatics. Hydration or structural forces of proteins under high salt conditions appear to play an important role.^35,36^

**Figure 3:**
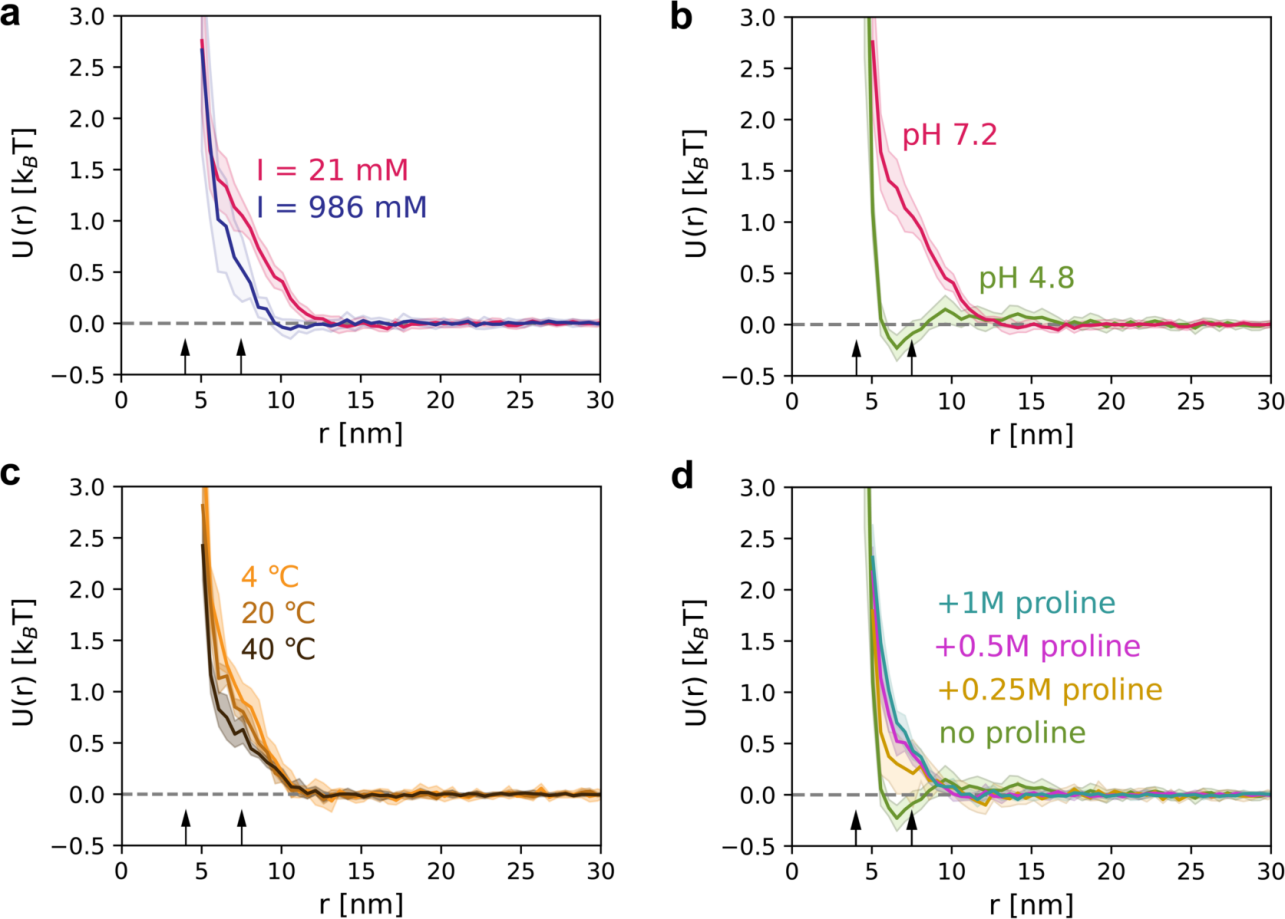
PIP of BSA under varied solution conditions: (a) ionic strength modulated with 10 mM or 975 mM NaCl while maintaining 5 mM phosphate buffer, pH 7.2; (b) pH varied at comparable ionic strength: 5 mM acetate buffer with 10 mM NaCl at pH 4.8 versus 5 mM phosphate buffer with 10 mM NaCl at pH 7.2; (c) temperature series at 4, 20, and 40 °C using 76 mM phosphate buffer at pH 7.2; (d) proline titration from 0 to 1 M at 5 mM acetate buffer with 10 mM NaCl at pH 4.8. BSA concentration was 20 mg/mL in all experiments; temperature was 20 °C except for c. The ionic strength was calculated by accounting for ionic species contributed by the buffer components and additionally added NaCl. Shaded areas indicate the standard deviation across replicates. The arrows indicate the smallest and highest dimensions of BSA as determined from its PDB structure.

We found that changes in pH lead to a stronger effect on the PIP than does ionic strength. For instance, at pH 4.8, close to the isoelectric point of BSA (pI ∼4.7–4.9),^37^ the measured PIP shows only a small ∼0.15 *k*_B_ *T* energy barrier, and at short distances, it tends toward an attractive primary minimum near the protein diameter, consistent with what is expected for protein systems under these conditions (Figure 3b).^38^

Furthermore, PIPs of proteins are known to depend on temperature. It is therefore important to examine the temperature dependence of protein interactions to understand the complex behavior of proteins under varying thermal conditions. For example, structural stability, functionality, and phase behavior are all significantly influenced by temperature. BSA is known to exhibit a lower critical solution point (LCST) in its phase diagram and undergo liquid–liquid phase separation (LLPS) at elevated temperatures.^39^ This LLPS behavior indicates a temperature dependence of intermolecular forces, where higher temperatures promote stronger attractive or lower repulsive interactions between BSA molecules, resulting in a net increase in attraction. This phenomenon has been demonstrated previously via *B*_22_ measurements by AUC-SE^40^ and by SAXS^16^ analysis.

We measured the temperature-dependent PIP of BSA at 4 °C, 20 °C, and 40 °C, as shown in Figure 3c. Our data show a clear trend: the net interactions between BSA molecules become increasingly attractive (or less repulsive) as the temperature rises. This observation aligns well with the known phase diagram of BSA, where elevated temperatures favor LLPS due to enhanced intermolecular attractions.^39,41^

Finally, our group has recently proposed a theoretical framework to explain the colloidal stabilization of general nanoscale dispersions by amino acids.^22^ According to our theory, the colloidal stabilization can be promoted through the screening of attractive interactions via weak binding of small molecules to the surface of the nanoscale colloids. The effect has been demonstrated with *B*_22_ measurements by AUC-SE. While analyses of interaction potentials were done with metallic-core Au nanoparticles and ferritin via their PMF, they have not yet been directly demonstrated for small globular proteins. Using the cryo-ET approach developed here, we quantify changes in the PIP of BSA upon the addition of proline near the isoelectric point, where attractive forces dominate due to the net-zero surface charge of the protein (Figure 3d). The addition of proline does not alter the protein surface charge, which remains net neutral under these conditions, as confirmed by zeta potential measurements (Table SI5). Nevertheless, proline induces a marked change in the PIP of BSA: the interactions become shorter-ranged (decay at 10 nm instead of 18 nm) and switch completely to a repulsive regime. At higher proline concentrations, further changes in the PIP are observed predominantly at distances below 8.5 nm, most likely caused by modifications to the hydration layer.^42^ Together, these results support the stabilization mechanism proposed in our theoretical framework.^22^

### PIP of Many Other Small Proteins

We applied our PIP measurement method to other globular proteins, including lysozyme, ovalbumin, streptavidin, hemoglobin, and human serum albumin (Figure 4a–e). As expected, within the concentration ranges used, the obtained PMF curves overlap, and the resulting PIP confirms that dilute-limit conditions were achieved for all proteins studied. The PIP resembles a typical interaction form of a soft sphere, i.e., a repulsive core with a finite interaction range that decays over a length scale of approximately two protein diameters. A comparatively steeper repulsion is observed for hemoglobin which may arise from its isoelectric point being close to the pH of the solution. Among the proteins used, lysozyme lies close to the lower size limit commonly considered for cryo-ET (example tomogram shown in Figure SI8).^43^ Previous cryo-ET reports on lysozyme have focused on nanocrystals;^44^ in this work, we include lysozyme to demonstrate the performance of our approach near this size regime. The method is equally applicable to larger proteins such as apoferritin; indeed, we demonstrate the PIP of apoferritin at pH values near its isoelectric point, where net-attractive interactions are observed (Figure 4f).

**Figure 4:**
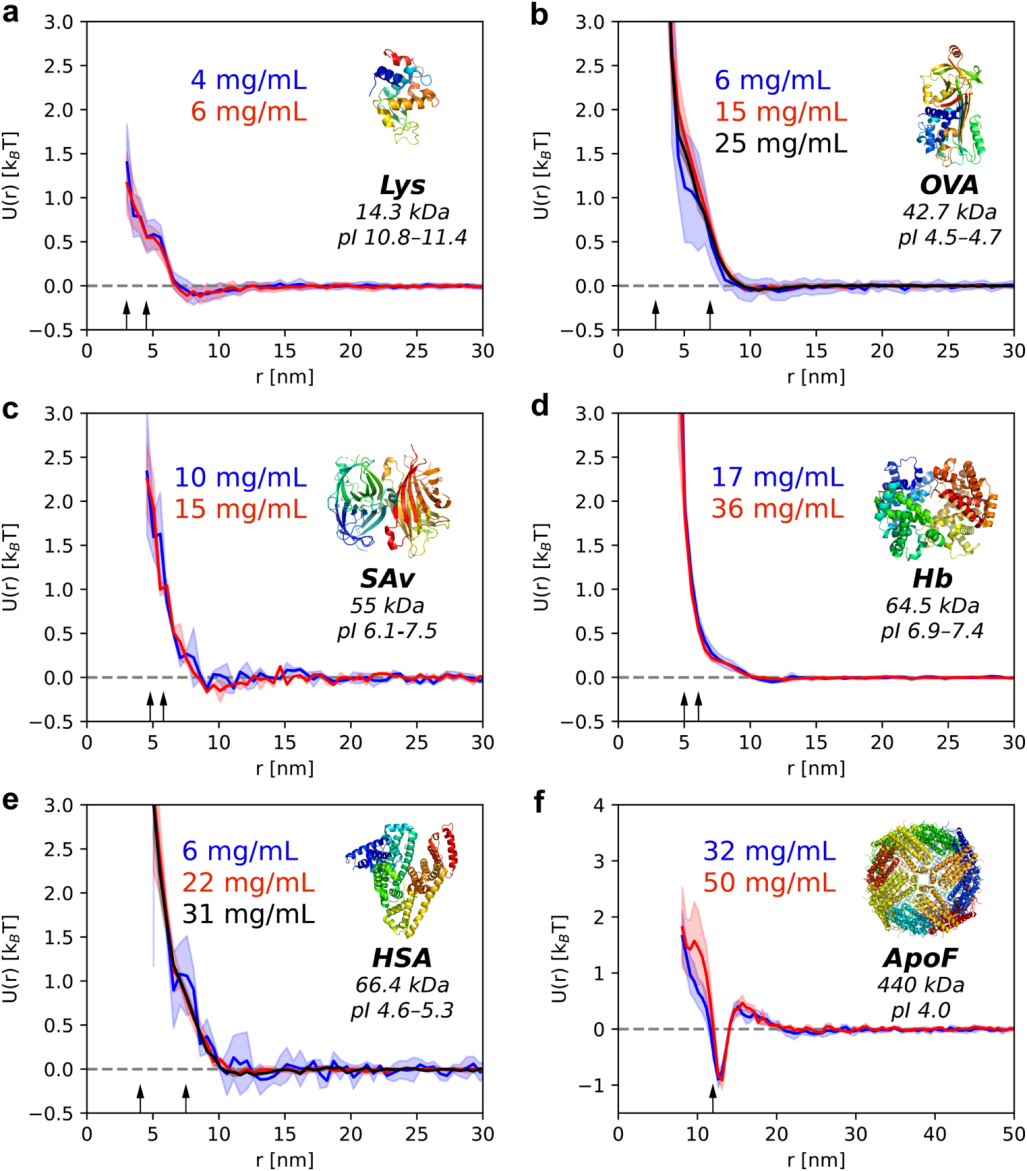
Dependence of the PIP on centroid-to-centroid distance for various globular proteins: (a) lysozyme, (b) ovalbumin, (c) streptavidin, (d) hemoglobin, (e) human serum albumin, (f) apoferritin. Buffer conditions: (a–e) 76 mM phosphate buffer, pH 7.2; (f) 10 mM acetate buffer, pH 5.0. All measurements were performed at 20 °C. Shaded areas indicate the standard deviation across replicates. The arrows indicate the smallest and largest molecular dimensions of proteins as determined from the PDB structures.

## Discussion

The workflow and results presented here establish cryo-ET as a powerful method for directly measuring protein thermodynamics across a wide size range—from small globular proteins at the very limit of tomographic resolution to large macromolecular assemblies. With only a few microliters of sample, this approach provides access to key thermodynamic quantities, including the pair interaction potential, the Kirkwood–Buff integral, and the structure factor. Thus, our method enables us to explore how PPIs are modulated by ionic strength, pH, temperature, and small-molecule additives. Our method offers a route toward the accumulation of multiple sets of data for proteins in many conditions, which can provide a fundamental basis for developing predictive theoretical models of protein interactions. This is particularly significant given that classical Derjaguin–Landau–Verwey–Overbeek (DLVO)-type models are known to be insufficient to account for several experimental observations, including the persistence of protein stability at high salt concentrations,^32^ as demonstrated here. As noted by others,^45,46^ we found additionally DLVO theory does not adequately model our experimental PIPs as presented in Figure SI9. The difference is more pronounced at short interparticle distances which can be attributed to the charge model and possibly the influence of anisotropic interactions that are not quantitatively determined from PIPs.

Several practical issues need to be considered when applying our cryo-ET method to protein solutions. Typical challenges include (1) protein adsorption to the AWI, (2) ice contamination at the AWI, (3) uneven ice thickness, and (4) sample tilt in the XZ and YZ planes (Figure SI1a). In this work, we solved the first three by analyzing only the central part of the tomogram and the last one by numerically tilting the particle coordinate array. Figure SI1b illustrates the result of this correction on segmented particles. Our validation using the orthogonal techniques confirms that the AWI does not affect the distribution of the proteins in the bulk of the tomogram. A fundamental limitation of the current approach, however, is that it yields an angular-averaged PIP; thus, any anisotropic (“patchy”) interactions on the protein surface are folded into an effective isotropic potential and cannot be assigned to specific sites. Further methodological developments to resolve orientation-dependent interactions are currently underway. Furthermore, our method works best for proteins that are stable in relatively high concentration and resistant to denature. Big proteins and proteins with high mass density would facilitate their detection in the image segmentation step. The field of cryo-ET has been progressing quickly, providing high-quality data sets at high throughput. Together with robust tomography grids, new generations of highly sensitive electron detectors, robot-based sample preparation, and continued software development, these advances will enable this approach to achieve a lower detection limit and increased sensitivity to weaker interactions.^24,47^

The rapid progress in *in situ* cryo-ET suggests that measurements of protein interaction potentials directly inside cells or tissues may become realistic in the near future, broadening the scope of our method.^48,49^ In the same vein, a possible application of our method to the analysis of interactions within biomolecular condensates could help clarify the mechanisms by which specific interactions drive phase separation.^50^

## Conclusion

In this work, we present a direct method for measuring the interaction potential of proteins using the cryo-ET technique. It was found that the potential of mean force measured for all small proteins studied here is weakly dependent on protein concentration and can therefore be considered an effective PIP. Importantly, this pair interaction potential is obtained, in particular, without assuming the analytical form or shape of the potential. In addition to the PIP, our method enables the measurement of other important thermodynamic parameters, such as the KBI and the structure factor. We successfully validated this approach with orthogonal methods such as AUC and SAXS. Moreover, this technique further enables systematic analysis of protein interactions under varying experimental conditions: increasing ionic strength partially screens repulsion, approaching the isoelectric point leads to weak attractions, elevated temperature makes interactions less repulsive in line with BSA’s LCST-type phase diagram, and small-molecule additives such as proline switch net-attractive interactions into repulsive ones. This method has the potential to become a new standard for the direct measurement of protein PIP.

## Materials and Methods

### Materials

Sodium phosphate monobasic (Sigma-Aldrich), sodium phosphate dibasic (Sigma-Aldrich), and Milli-Q water were used to prepare phosphate buffer (76 mM, pH 7.2). All other reagents were purchased from Sigma-Aldrich unless otherwise stated. Gibco PBS 1× was purchased from Thermo Fisher Scientific. Bovine serum albumin (BSA) human serum albumin (HSA), ovalbumin (OVA), human hemoglobin (Hb), and apoferritin from equine spleen (ApoF) were obtained commercially. Streptavidin (SAv) was from AppliChem, and lysozyme (Lys) from Roche. Protein physicochemical properties, including molecular weight, dimensions, hydrodynamic diameter, and isoelectric point, are summarized in Table SI7. Further information about the sample conditions is provided in Table SI8.

### Methods

#### Protein Fractionation by Fast Protein Liquid Chromatography (FPLC)

The HiLoad 26/600 Superdex 200 PG column was equilibrated with PBS 1×. Two milliliters of 40 mg/mL protein solution were injected into an ÄKTA go FPLC system (Cytiva) at 1.3 mL/min and 4 °C. The monomeric fraction was collected and concentrated in Amicon Ultra centrifugal filters (MWCO 3 or 10 kDa) at 5000 rpm and 4 °C. Buffer exchange was achieved through iterative dilution and reconcentration steps in freshly prepared phosphate buffer. The filtrate from the fifth concentration cycle was used as the matching background buffer in subsequent experiments.

#### Analytical Ultracentrifugation (AUC)

AUC–sedimentation velocity (**AUC–SV**) experiments were performed to verify protein sample purity using an Optima XL-I analytical ultracentrifuge (Beckman Coulter) equipped with absorbance optics at 280 nm. Protein samples (376 μL) with absorbance values between 0.2 and 1 were loaded into 12 mm double-sector cells, with 380 μL phosphate buffer in the reference sector. Runs were conducted at 20 °C with a radial step size of 0.003 cm, and data were collected in continuous scan mode. Data were analyzed in SEDFIT^51^ using maximum-entropy regularization at a confidence level of 0.68 and an s-resolution of 200 over 0–15 S.

AUC–sedimentation equilibrium (**AUC–SE**) experiments were used to determine second virial coefficients following a published procedure.^52^ Samples (∼10 mg/mL) were loaded into 3 mm path length cells, and runs were performed at 20 °C using interference optics. The protein concentration gradient at the sedimentation–diffusion equilibria with a depleted meniscus was obtained and converted into the equation of state Π(ρ) curve where Π is the osmotic pressure and ρ is the protein number density. The second virial coefficient was found as the slope of the $\frac{\Pi }{\rho }\left(\rho \right)$ curve.

#### Cryogenic Electron Tomography (Cryo-ET)

Protein solutions were applied to the original Chameleon foil grids (300 mesh, R1.2/0.8, SPT Labtech, UK) using a Chameleon system (SPT Labtech, UK) in single-stripe mode with a plunge time of 301 ms under 80% humidity at 20 °C after 60 s of online glow discharge at 12 mA. For temperature-dependent experiments, copper 200-mesh R1.2/1.3 Quantifoil grids were glow-discharged for 90 s at 15 mA, after which both the protein sample and the Vitrobot Mark IV (Thermo Fisher Scientific) chamber were pre-equilibrated to the desired temperature at 100% humidity. Four microliters of sample were applied to each grid, blotted from both sides for 1 s, and immediately plunge-frozen into liquid ethane. Imaging was done at the Dubochet Center for Imagine (Lausanne, Switzerland) using Titan Krios G4 (Thermo Fisher Scientific) operated at 300 kV. Tilt series were acquired using a dose-symmetric scheme with ±40° tilt range and 2° increments (see Supporting Information, Figure SI10, for details) at 42,000× magnification (pixel size 0.3 nm), −7 μm defocus, and a total dose of 100 e^–^/Å^2^. Images were recorded on a Falcon 4i camera equipped with a Selectris X energy filter (10 eV slit width). Prealigned MRC files were compiled from Tomography v.5.16.0 (Thermo Fisher Scientific) and were used for the image analysis.

#### Image Analysis

Tilt series (binned by a factor of 2; and by a factor of 3 for apoferritin) were aligned mainly in IMOD^53^ (with a few checked in Inspect3D, Thermo Fisher Scientific) using cross-correlation followed by patch tracking (patch size 2000 × 2000 px, overlap 0.33, trim 500 px, high-frequency cutoff 0.01), ensuring a residual alignment error below 0.4 nm. Tomographic reconstructions were performed in IMOD using the Simultaneous Iterative Reconstruction Technique (SIRT) with 20 iterations. The resulting tomograms were contrast-inverted in Fiji^54^ (National Institutes of Health, USA) prior to segmentation in Imaris v.10 (Bitplane, Spots module). For spot detection, the XY diameter was derived from 2D slices, and Z elongation (due to the missing wedge) was fixed at 1.3× the XY diameter. Typical reconstructed volumes were ∼800 × 800 × 100 nm^3^. The 3D positions of protein macromolecules were extracted and scaled by the calibrated pixel size of 0.3 nm. For lysozyme, a 3D Gaussian blur (σ = 0.75) was applied to the tomograms using Fiji prior to segmentation to reduce noise. In the case of hollow apoferritin, segmentation was performed on noninverted tomograms, targeting the empty core of the protein. Missing-wedge artifacts were reduced using an IsoNet neural network,^55^ trained and applied prior to segmentation. A SegWiz U-Net with depth level 3 and initial filters count of 64 model was trained in Dragonfly^56^ on one tomogram (10 annotated slices) to classify background, empty core, protein shell, and defocus-induced shell (Figure SI11). Final centroids were obtained in Imaris (Bitplane) via Surface detection with the spot-splitting option.

#### Calculations of RDF, KBI, and *S*(*q*) from Particles 3D Coordinates

Preparation of the final array of 3D protein coordinates (including tilting and cropping) was performed using in-house-developed Python code. The Python code for RDF calculation^57^ was rewritten in the Julia programming language, significantly reducing the computation time. The RDF was computed with a bin size of 0.3 nm (corresponding to the tomogram pixel size) and 100 bins. The KBIs were obtained using custom Julia routines (theoretical background is described at the end of this section). Structure factors were calculated from the particle positions in the tomograms using MATLAB scripts implementing a histogram-based algorithm. All scripts were integrated into an interactive Pluto.jl notebook and are publicly available in Github (https://github.com/epoliukhina/Potential.git).

#### SAXS Measurement

SAXS measurements were performed on a small-angle X-ray diffractometer Xeuss 3.0 (Xenocs, France) at ETH (Zurich, Switzerland) using an X-ray Cu source (wavelength Kα 1.5418 Å). Measurements were carried out at room temperature in moderate vacuum conditions to avoid air scattering. The sample-to-detector distance (300 mm) was calibrated with a standard silver behenate powder. All protein samples were prepared in a quartz capillary with a diameter of 1.5 mm (Hilgenberg Company, Germany). The scattering frames of the protein sample and the proper buffer were recorded in the same conditions. The buffer data were considered as background and subtracted before data analysis with preliminary corrections for capillary diameter discrepancy. Different protein concentrations were investigated. The background-subtracted *I*(*q*) of the sample with the lowest measured concentration in (1.25 or 5 mg/mL) was to represent the form factor, *P*(*q*), since there was no increase in forward scattering for this sample and showed a plateau in small q range indicating the negligible interaction. The *P*(*q*) curve was fitted with a triaxial ellipsoid model in SasView software. The structure factors *S*(*q*) were calculated by dividing the *I*(*q*) by fitted *P*(*q*) and normalized to 1 at high *q*.

#### Zeta Potential Measurement

Zeta potential (ZP) was measured using a Litesizer 500 (Anton Paar, Austria).

The refractive index and viscosity of each buffer were determined using an Abbe refractometer (ATAGO, Japan) and a Lovis 2000 ME microviscometer (Anton Paar), respectively.

#### Extraction of KBI from Cryo-ET Tomograms of Proteins

The quantitative description of PPIs can be given in the form of interaction potential, which is, by general definition, the work required to bring two protein macromolecules from an infinite distance to a finite distance.^11^ There exist two forms of interaction potential: the potential of mean force (PMF, *W*(*r*)) and the pair interaction potential (PIP, (*U*(*r*)), which are linked with each other through the following equation:

1 W(r)=U(r)+Δw(r)

where Δ*w*(*r*) is the correction taking into account the effect of surrounding particles, *r* is the interparticle centroid-to-centroid distance. In other words, the concentration-dependent *W*(*r*) converges to the concentration-independent *U*(*r*) at the infinite dilution limit.^11^ In statistical-mechanical theory, the PMF can be found through the reversible work theorem:

2 $$W\left(r\right)=-k_{B}T\ln\left[g\left(r\right)\right]$$

where *g*(*r*) is the radial distribution function (RDF) which can be directly calculated if the spatial distribution of particles is known.^58^

Another thermodynamic quantity that can provide insight into PPIs is the second virial coefficient *B*_22_ and the Kirkwood–Buff integral (KBI, $G_{22}^{\infty }$ ):

3 $$B_{22}=-2\pi \int_{0}^{\infty }\left(e^{-\frac{U\left(r\right)}{k_{B}T}}-1\right)r^{2}dr$$

4 $$G_{22}^{\infty }=4\pi \int_{0}^{\infty }\left(e^{-\frac{W\left(r\right)}{k_{B}T}}-1\right)r^{2}dr$$

where *k*_B_ is the Boltzmann constant and *T* is the Kelvin temperature. One can see (eqs 1, and 3,4) that at the dilute regime reached for proteins, the *B*_22_ and KBI are proportional by a factor of −2:

5 $$G_{22,0}^{\infty }=-2B_{22}$$

The particle spatial distribution readily available from cryo-ET tomograms suggests that one can apply statistical treatment to extract useful thermodynamic parameters.^11^ Here, we use cryo-ET to determine the KBI of protein systems in several ways. First, by the statistical-mechanical definition, the KBI can be obtained by direct integration of the RDF (Figure SI6a):

6 $$G_{22}^{\infty }=4\pi \int_{0}^{\infty }\left(g\left(r\right)-1\right)r^{2}dr$$

The cumulative *G*_22_(*r*) for BSA, computed by replacing the upper limit with *r* in eq 6, is shown in Figure SI6b. Because *g*(*r*) starts its fluctuation around 1 at a distance of approximately 18 nm, *G*_22_(*r*) is expected to reach its asymptote $G_{22}^{\infty }$ near that distance. This, however, is only partially observed, revealing two main disadvantages of this approach: 1) the experimentally found *g*(*r*) is a noisy function, and noise being multiplied by *r*_2_ results in an enormous noise impact when integrating, and 2) in a finite-size box system, calculations may lead to the RDF not converging to 1.^40^ The second issue is mainly solved using the advanced RDF calculation method.^57^ However, the “noise” problem is clearly present, as evidenced by the growing deviation between replicates at the distances of 15 nm and higher.

To circumvent this drawback, we apply the so-called sub-box (Schnell’s) method,^31–33^ a recently developed statistical mechanics method for samples of finite volume. This method uses the density fluctuations theory, which states that in the grand-canonical ensemble (μ,*V*,*T* = const), the KBI can also be rewritten as

7 $$G_{22}=\frac{V}{{\langle N_{2}\rangle }^{2}}\left(\left\langle N_{2}^{2}\right\rangle -{\langle N_{2}\rangle }^{2}-\left\langle N_{2}\right\rangle \right)$$

where *V* is the volume of the sub-box, ⟨*N*_2_⟩ is the ensemble-averaged number of particles in the sub-box. Practically, it is performed by dividing the cryo-ET tomogram (“bath” in statistical-mechanical terms) into sub-boxes—which can exchange particles and energy with the “bath”—of the chosen geometrical shape (Figure SI6c). In our case, the sub-boxes are parallelepipeds with a square base of side *L* and fixed height of the tomogram *H*. Sub-boxes overlap with a user-chosen step. Decreasing the step smooths the resulting curve without changing its trend (Figure SI6d); accordingly, we fix the step at 3 nm for all analyses. By conducting this division for sub-boxes of various sizes, one can determine the appropriate value of $G_{22}^{\infty }$ . This is achieved when the volume of the sub-box is sufficiently large so that *G*_22_ becomes independent of the size of the box. In contrast to gold nanoparticles, which exhibit a clear plateau with increasing sub-box size,^19^ proteins do not: the effective KBI monotonically decreases with *L* (Figure SI6e). We found that the robust approach, in this case, is to determine KBI as the intercept of the dependence of effective *G*_22_ on surface area-to-volume ratio $\frac{S}{V}$ of a sub-box (Figure SI6f):

8 $$G_{22}\left(\frac{S}{V}\right)=G_{22}^{\infty }+\frac{S}{V}\times G_{22}^{\prime }$$

where *S* is the surface area of the sub-box, $G_{22}^{\prime }$ is the surface contribution of *G*_22_.

The linear regime of $G_{22}\left(\frac{S}{V}\right)$ corresponds to *L* values below 50 nm.

## Supplementary Material





## Notes

Workflow comparison for AuNPs and proteins; Postprocessing of segmented coordinates; Protein FPLC purification and *c*(*s*) distribution measured by AUC-SV; Statistical comparison of PMF profiles at different protein concentrations; Results of the SAXS measurements; Results of the *B*_22_ measurements by AUC-SE; Extraction of KBI from cryo-ET tomograms of proteins; Determination of the *B*_22_ by fitting the concentration dependence of *S*(*q* = 0); Results of the zeta potential measurements; Tomogram of lysozyme and its segmentation; Fitting of BSA *U*(*r*) using the DLVO equation; Protein *c*(*s*) distribution measured by AUC-SV; Effect of the tilt-series parameters on the PMF; Segmentation of apoferritin (PDF)
Video showing the reconstructed tomogram of BSA followed by segmentation results (MP4)
