# Supplementary material for: Direct Measurement of Protein Pair Interaction Potential
Source: ACS Nano. 2026 Mar 6;20(10):8487–97. doi: 10.1021/acsnano.5c19213 (PMC13001087; doi:10.1021/acsnano.5c19213)
Supplement: Supplementary file 1 [file nn5c19213_si_001.pdf]

SUPPORTING INFORMATION

Direct Measurement of Protein Pair Interaction Potentials

Ekaterina Poliukhina[1], Quy Ong[1], Davide Demurtas[2], Emiko Uchikawa[3], Notash Shafie[1], Francesco Stellacci [1,4,5*]*

[1]Laboratory of Supramolecular Nanomaterials and Interfaces, Ecole Polytechnique Fédérale de Lausanne (EPFL), 1015, Lausanne, Switzerland.

[2]Interdisciplinary Centre for Electron Microscopy (CIME), Swiss Federal Institute of Technology Lausanne (EPFL), 1015, Lausanne, Switzerland.

[3]Dubochet Center for Imaging (DCI), Ecole Polytechnique Fédérale de Lausanne (EPFL) and University of Lausanne, 1015, Lausanne, Switzerland.

[4]Institute of Bioengineering, École Polytechnique Fédérale de Lausanne (EPFL), Station 12, 1015, Lausanne, Switzerland.

[5]Global Health Institute, École Polytechnique Fédérale de Lausanne (EPFL), Station 12, 1015, Lausanne, Switzerland.

Corresponding authors: Quy Ong quy.ong@epfl.ch

Francesco Stellacci francesco.stellacci@epfl.ch

Table of Contents

1. Workflow comparison for AuNPs and proteins 3

2. Post-processing of segmented coordinates 5

3. Protein FPLC purification and c(s) distribution measured by AUC-SV 7

4. Summary of tomograms used in this work 9

5. Statistical comparison of PMF profiles at different protein concentrations 10

6. Results of the SAXS measurements 12

7. Results of the B_{22} measurements by AUC-SE 13

8. Extraction of KBI from cryo-ET tomograms of proteins 14

9. Determination of the B_{22} by fitting the concentration dependence of $S(q = 0)$ 15

10. Results of the zeta potential measurements 16

11. Tomogram of lysozyme and its segmentation 17

12. Fitting of BSA U(r) using DLVO equation 18

13. Summary on proteins and sample conditions 19

14. Effect of the tilt-series parameters on the PMF 21

15. Segmentation of apoferritin 23

1. Workflow comparison for AuNPs and proteins

Table SI1. Cryo-ET methods. Comparison of parameters in the Cryo-ET workflow of our published method for AuNPs (Ong, Q. *et al.* Mater. Horiz. 2022) and for proteins (current work).

	**Parameter**	**AuNPs (Mater. Horiz., 2022)**	**Proteins (this work)**	**Remarks**
Material properties in electron microscopy				
1	Contrast	High	Low	Imaging with high defocus values improve image contrast. This parameter is crucial for protein samples.
2	Interfacial activity	Absent	From middle to high	Adsorption of proteins at the air-water interface (AWI) and their following denaturation/aggregation is common. We use high concentration of protein. Highly surface-active proteins saturate the AWI, providing homogeneous protein concentration in the bulk of the sample.
3	Detectability	Easy	From middle to high	AuNPs have high contrast, while proteins in general have much lower contrast, and such difficult to image.
4	Susceptibility to freezing	Low	High	Proteins are more susceptible to freezing condition in the conventional paper-based blotting. It is mitigated by blot-free robotized Chameleon technique.
5	Concentration used	Low	High	For proteins, we tend to use much high concentration of sample to saturate the AWI (see 2).
6	Polydispersity	Yes	No	To obtain PIP of proteins, it is crucial to remove oligomers and large aggregates. We utilize rigorous FPLC purification.
7	Aggregate detection	Easy	Challenging	Because of the low contrast of small proteins (MW <100KDa). In this work, only monomers of proteins are used (see 6)
8	Ice thickness	Up to 300 nm	100–200 nm	For proteins, we tend to obtain medium thickness of our samples. It is a compromise between particle counts and contrast for proteins.
9	Beam dose sensitivity	Low	High	For proteins, we apply limitation of total dose to $120e^-$ to protect integrity of proteins
Post-processing step				
10	Tomogram tilt	Not applied	Digital alignment using 3D coordinates	Tomograms often have the Z slices being not parallel to the XOZ plane. In AuNPs work, we did not correct for this tilt which in some cases led to erroneous PMF because of its numerical

				normalization. In this work, we performed a tilt correction after extraction of particle positions. Possible errors due to non-uniform distribution and edge tilt are removed.
11	Computation cost		Simple iterative Julia language: fast output	In AuNPs work, python and Matlab codes are used. In this work, the treatment of our 3D data is done in Julia.
Extracted thermodynamic quantities				
12	PMF and KBI	Concentration dependent	Concentration independent	In AuNPs work, we obtain PMF and Kirkwood-Buff integrals G_{22} by classical box method. The PMP and G_{22} are concentration dependent, which is not the case for small protein in this work. This difference, probably, is due to strong attractive forces between AuNPs and much weaker interactions between proteins.
Microscope setup				
13	Acceleration voltage	200 keV	300 keV	We found the electron microscope in our AuNP work (200keV, Falcon III) not suitable to image proteins for the purpose of the current work. The new instrument (equipped with 300keV, Selectris energy filter, Falcon4i, sample cartridge) substantially improves the throughput and tomogram quality.
14	Energy filter	none	Selectris energy filter, slit of 10 eV	
15	Camera	Falcon III	Falcon4i	
16	Acquisition software	Tomo 2	Tomo 5	The later version provides much higher throughputs by providing high-speed image acquisition based on beam shift, and allows for on-the-fly tomogram reconstruction by *TomoLive.*

2. Post-processing of segmented coordinates

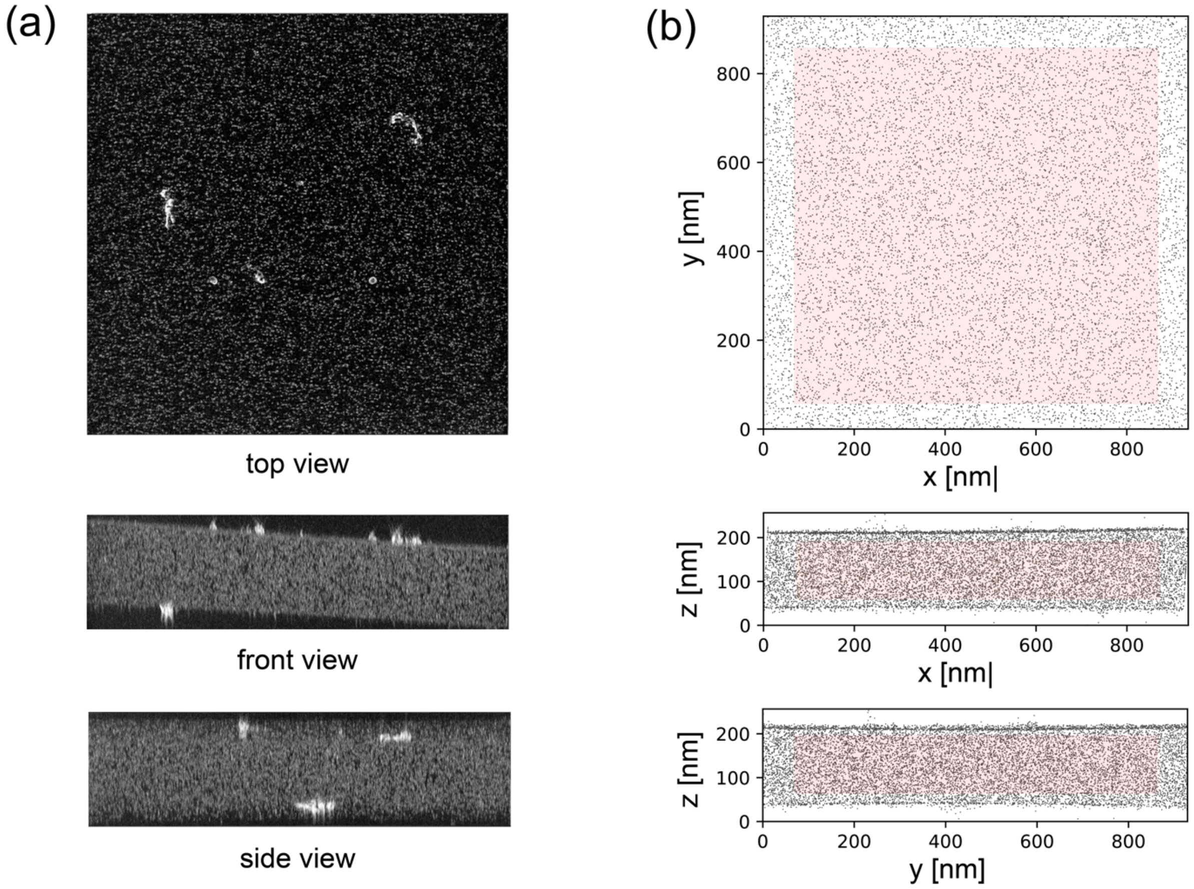

Figure SI1. Example of the tomogram of 10 mg/mL BSA in phosphate buffer shown as three projections **(a)** and corresponding segmented particles after tilting **(b)**. The highlighted area indicates the particles used for RDF calculation. The effect of tilt correction and cropping on particle uniformity and RDF analysis is summarized in **Table SI2**.

Table SI2. Effect of tilt correction and cropping on particle uniformity and RDF analysis.

	Case 1	Case 2	Case 3<br>(**used in this work**)
Post-processing	No tilt correction, no cropping	No tilt correction, only cropping	Tilt correction + cropping
Projection image	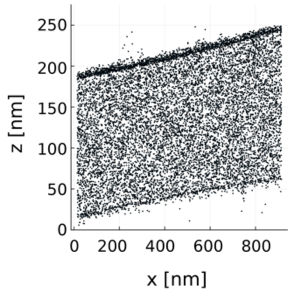 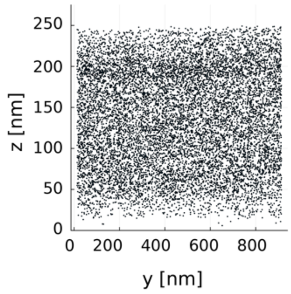 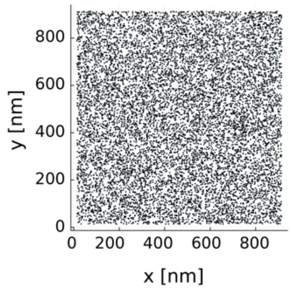	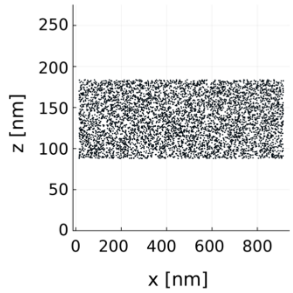 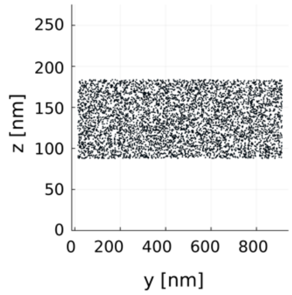 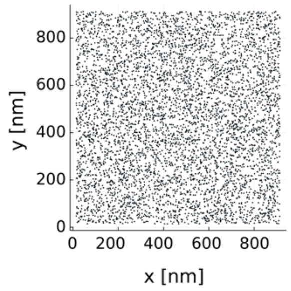	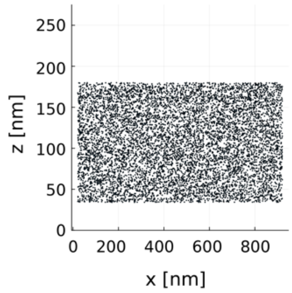 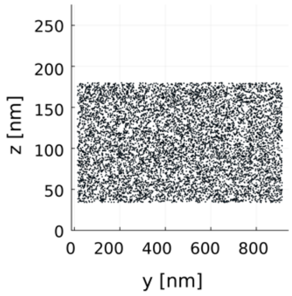 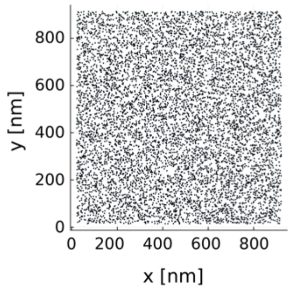
RDF plot	Doesn't converge to 1 at high *r* 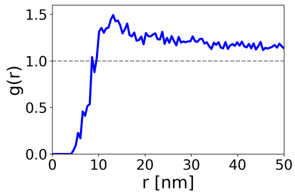	Converges to 1 at high *r*, but noisy 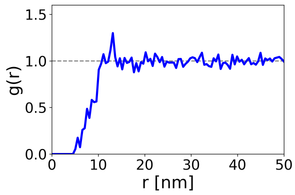	Converges to 1 at high *r* and less noisy 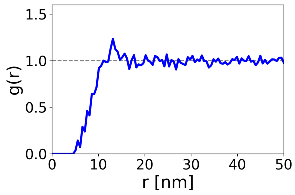
Comment	Non-uniform concentration, empty voids.	~35% of particles lost; depends on tilt.	Best uniformity, use of all data, and correct normalization.

3. Protein FPLC purification and c(s) distribution measured by AUC-SV

Our fast protein liquid chromatography (**FPLC**) step is used to remove pre-existing, irreversible high-molecular-weight species present in the commercial proteins preparation and to start from a well-defined monomeric population. Importantly, this purification does not suppress the dynamic, non-covalent near-contact associations that arise from the protein–protein interactions (**PPIs**) we quantify by cryo-ET.

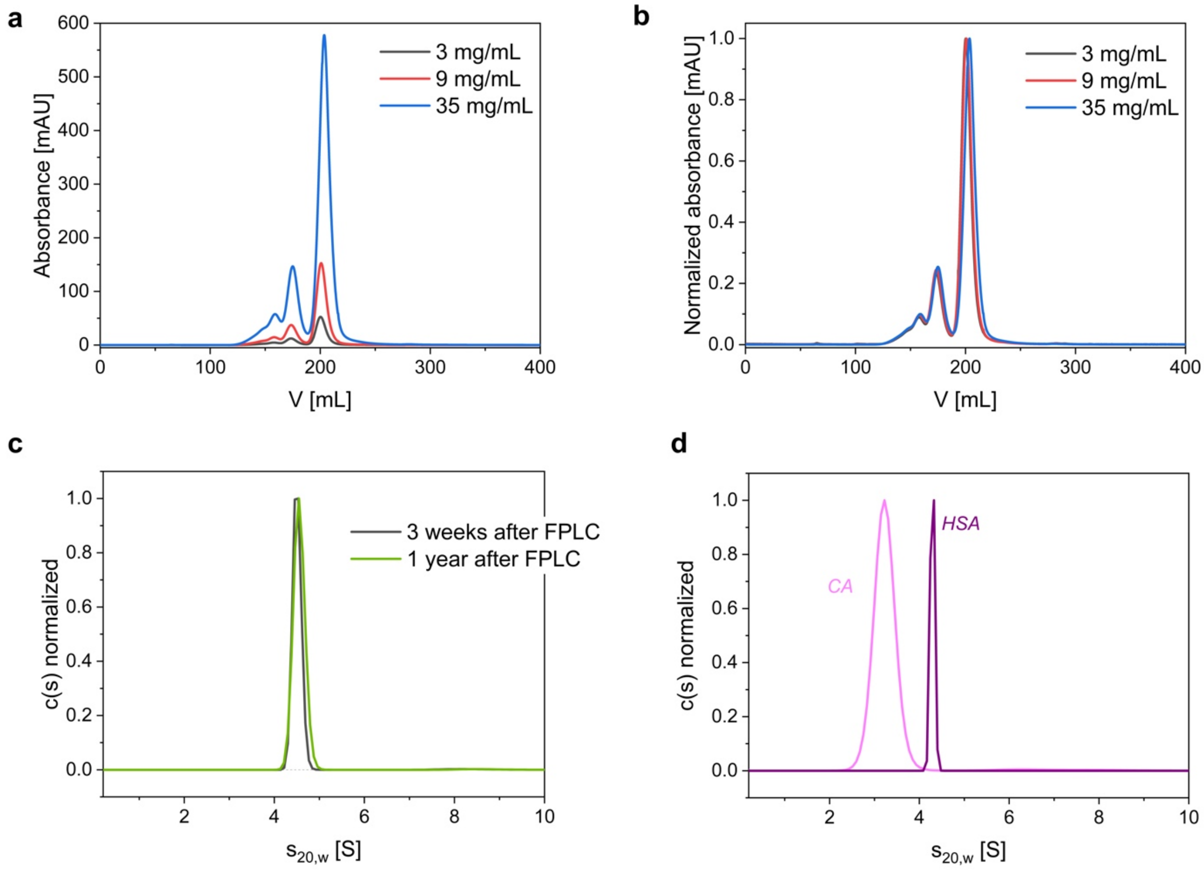

Figure SI2. (a) The chromatograms of BSA with raw UV absorbance (mAU) as a function of elution volume V for protein solutions at 3, 9, and 35 mg/mL in PBS1×, pH 7.4. **(b)** The same chromatograms normalized to their respective maximum absorbance to compare peak positions and profile shape independent of concentration. **(c)** Sedimentation velocity AUC c(s) distributions of monomeric BSA measured 3 weeks and 1 year after two rounds of FPLC fractionation. Measurements were performed in 76 mM phosphate buffer (pH 7.2) at 20 °C. **(d)** The c(s) distributions of CA and HSA used in this study. Measurements were performed in 76 mM phosphate buffer, pH 7.2, at 20 °C.

For instance, commercial bovine serum albumin (**BSA**, Sigma Aldrich) is known to contain monomer as well as disulfide-linked oligomers. In our sample, we detect ~72%

monomer, ~21% dimer, ~7% trimer and higher species. To test whether these oligomers are covalently bound rather than formed by reversible PPIs, we performed FPLC at three different injected concentrations and compared the normalized chromatograms (**Fig. SI2a**). The relative peak areas (mono/di/tri) are unchanged upon dilution (**Fig. SI2b**). If the dimer/trimer peaks originated from reversible association governed by the same PPIs measured by cryo-ET, the oligomer/monomer ratio would be expected to decrease upon dilution. The observed concentration-independence is therefore consistent with covalently linked (disulfide-bonded) species rather than non-covalent aggregates.

After isolating monomeric BSA, we find it is stable against re-formation of covalent dimers: AUC-SV shows no detectable emergence of covalent dimer even after 1 year of storage of 20 mg/mL monomeric BSA in 76 mM phosphate buffer at 4 °C (**Fig. SI2c**).

Crucially, removing these covalent/irreversible species does not eliminate near-contact PPIs contributions because the near-contact part of the potential is governed by transient encounters among monomers in solution at the measurement conditions. This dynamic non-covalent dimerization can still occur and is exactly what our cryo-ET-based PIP analysis captures (e.g. in the case of pH being close to the isoelectric point).

Therefore, the FPLC purification removes permanent/irreversible species that would otherwise confound the analysis by introducing a mixed population with different sizes/shapes and potentially different interaction propensities. The measured PIP reflects the equilibrium transient associations among monomeric proteins under the experimental conditions.

Table SI3. The composition of protein samples measured by AUC-SV. The integration of the peaks in **Fig. SI2c,d** was performed using the SEDFIT software.

	Monomer fraction, %	Dimer fraction, %	Trimer fraction, %
BSA	97.7 *(4.3S)*	1.9 *(7.2S)*	0.4 *(10.7S)*
CA	97.7 *(3.2S)*	1.9 *(6.9S)*	0.4 *(11.9S)*
HSA	99.4 *(4.2S)*	0.1 *(6.9)*	*0.5 (11.8S)*

4. Summary of tomograms used in this work

Table SI4. Summary of tomograms and particle counts used for RDF calculations across all datasets.

Dataset	Number of tomograms	Total number of particles for RDF	Fig. in the maintext
BSA, 4 mg/mL	5	12,431	
BSA, 8 mg/mL	6	27,527	2
BSA, 16 mg/mL	5	43,910	
BSA, pH 7.2, I = 21 mM	8	52,914	3a,b
BSA, pH 7.2, I = 986 mM	9	63,145	3a
BSA, pH 4.8, I = 15 mM	8	43,799	3b,d
BSA, 4 °C	5	27,059	
BSA, 20 °C	4	32,978	3c
BSA, 40 °C	5	45,324	
BSA, pH 4.8, 0.25M proline	5	23,323	
BSA, pH 4.8, 0.5M proline	6	63,604	3d
BSA, pH 4.8, 1M proline	6	64,124	
Lys, 4 mg/mL	11	79,227	4a
Lys, 6 mg/mL	9	73,517	
OVA, 6 mg/mL	11	60,024	
OVA, 15 mg/mL	18	227,381	4b
OVA, 25 mg/mL	13	279,831	
SAv, 10 mg/mL	3	11,435	4c
SAv, 15 mg/mL	3	15,844	
Hb, 17 mg/mL	5	64,315	4d
Hb, 36 mg/mL	5	77,275	
HSA, 6 mg/mL	3	9,659	
HSA, 22 mg/mL	4	50,830	4e
HSA, 31 mg/mL	2	36,350	
ApoF, 32 mg/mL	4	7,652	4f
ApoF, 50 mg/mL	3	15,398	

5. Statistical comparison of PMF profiles at different protein concentrations

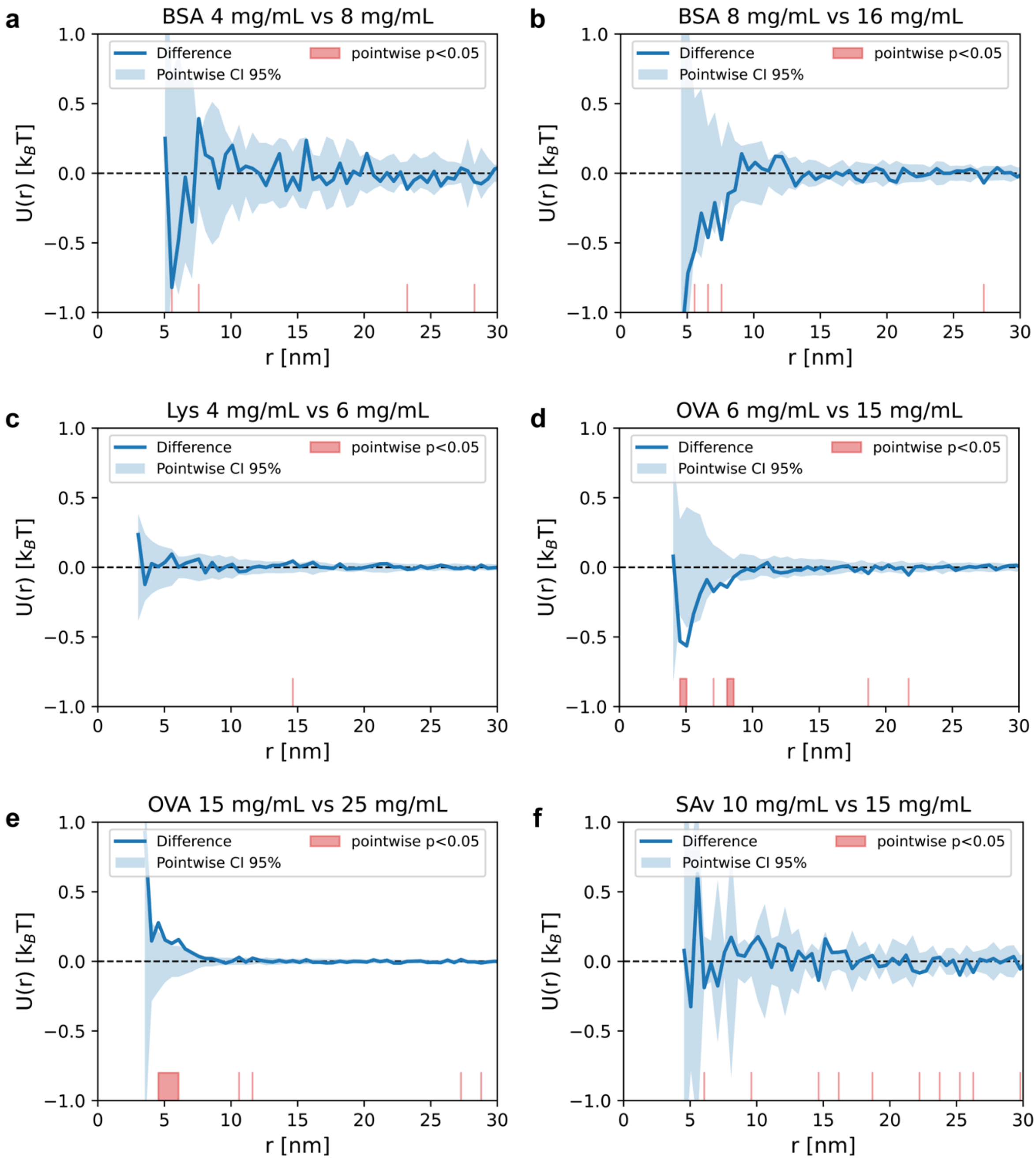

Figure SI3. Statistical comparison of PMF profiles at different protein concentrations: **(a, b)** BSA, **(c)** lysozyme, **(d, e)** ovalbumin (OVA), **(f)** streptavidin (SAv), **(g)** hemoglobin (Hb), **(h, i)** HSA, **(j)** apoferritin (ApoF). The blue solid line shows the difference between the averaged PMF curves, and the light-blue band is the pointwise 95% confidence interval for the difference, computed from replicate standard deviations using Welch t-intervals with the sample sizes indicated in **Table SI4**. Red shading marks r-regions where $|t(r)|$ exceeds the critical value (two-sided, α=0.05). At small distance, the difference gets larger even though at a small magnitude. We attribute this effect to the low counts of

number of particles as the circular volume used for RDF calculation is smaller, and the signal to noise consequently decreases steeply. Furthermore, an added uncertainty is expected to have larger effect for samples of lower concentration due to the drop in total number of particles counted.

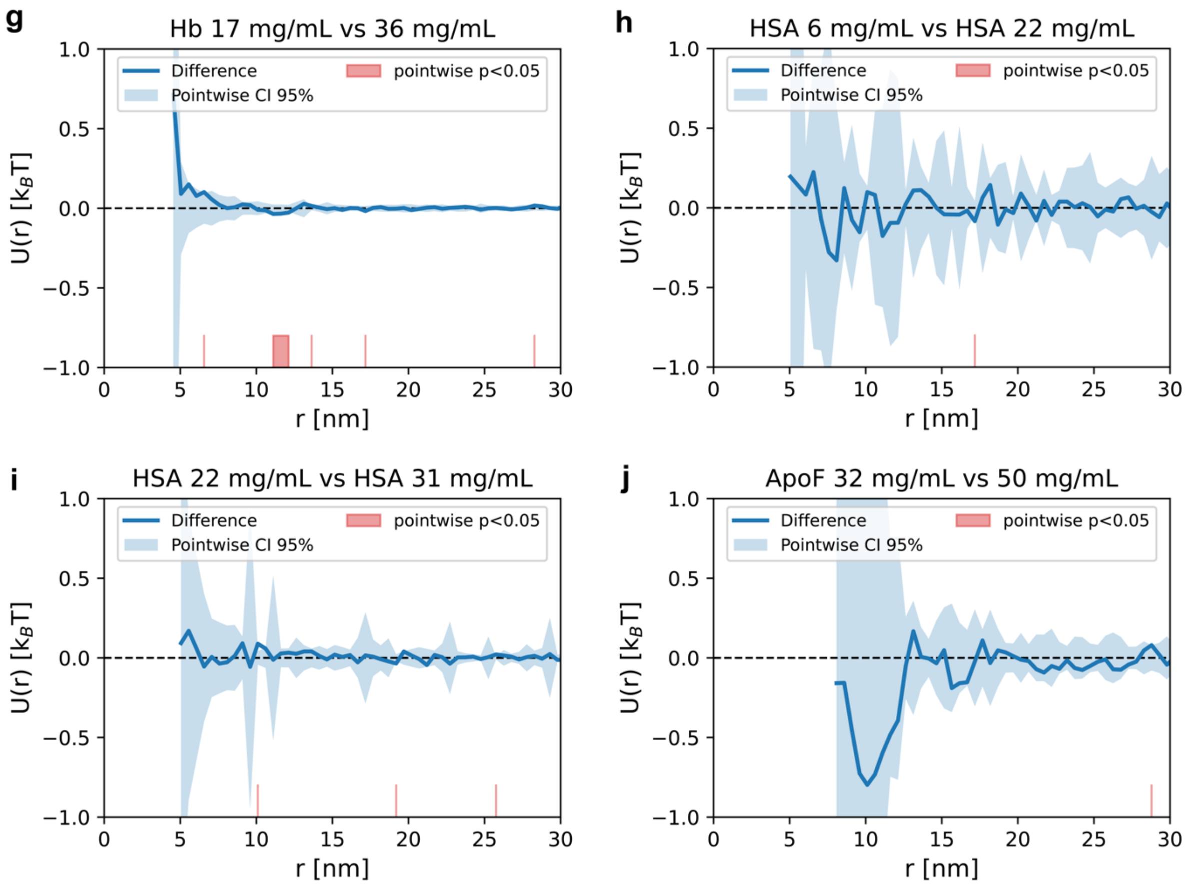

Figure SI3 (continued).

6. Results of the SAXS measurements

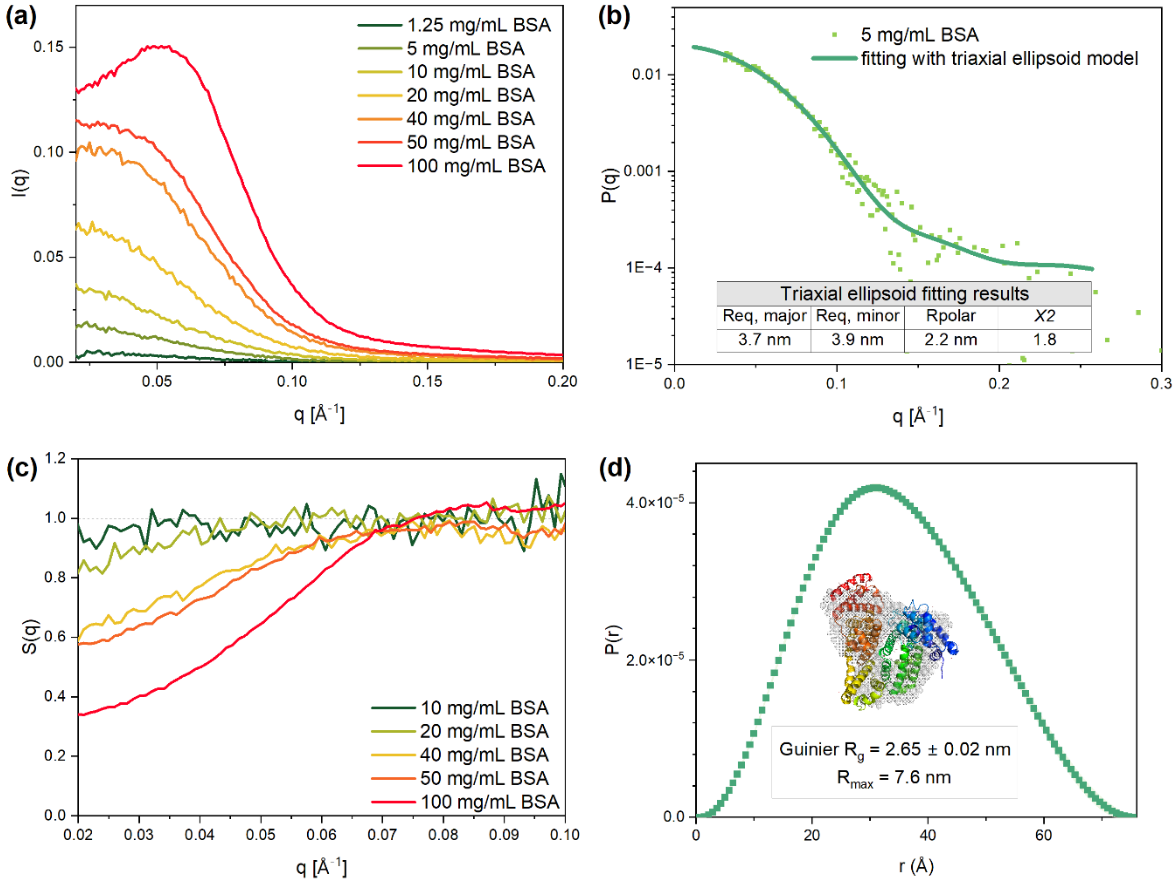

Figure SI4. The results of the SAXS experiment for BSA: background-subtracted scattering intensities $I(q)$ **(a)**; fitting of scattering intensity of 5 mg/mL sample taken as $P(q)$ by triaxial ellipsoid model and fitted parameters **(b)**; structure factor $S(q)$ **(c)**; distance distribution $P(r)$ and measured Guinier radius **(d)**.

7. Results of the B_{22} measurements by AUC-SE

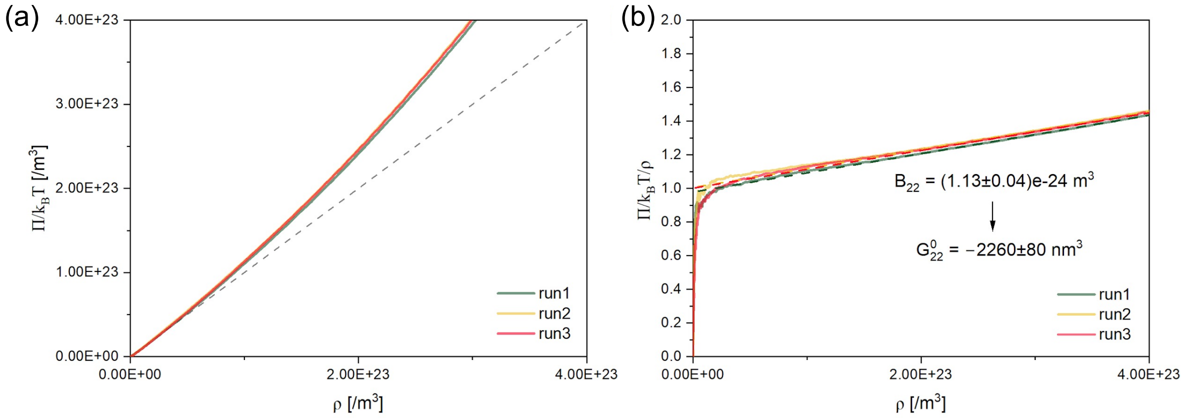

Figure SI5. Results of the AUC-SE experiment for BSA: **(a)** dependence of osmotic pressure on protein number density; **(b)** dependence of specific osmotic pressure on protein number density. The slope of the linear region in **(b)** yields the second virial coefficient. Measurements were performed in 76 mM phosphate buffer, pH 7.2, at 20 °C.

8. Extraction of KBI from cryo-ET tomograms of proteins

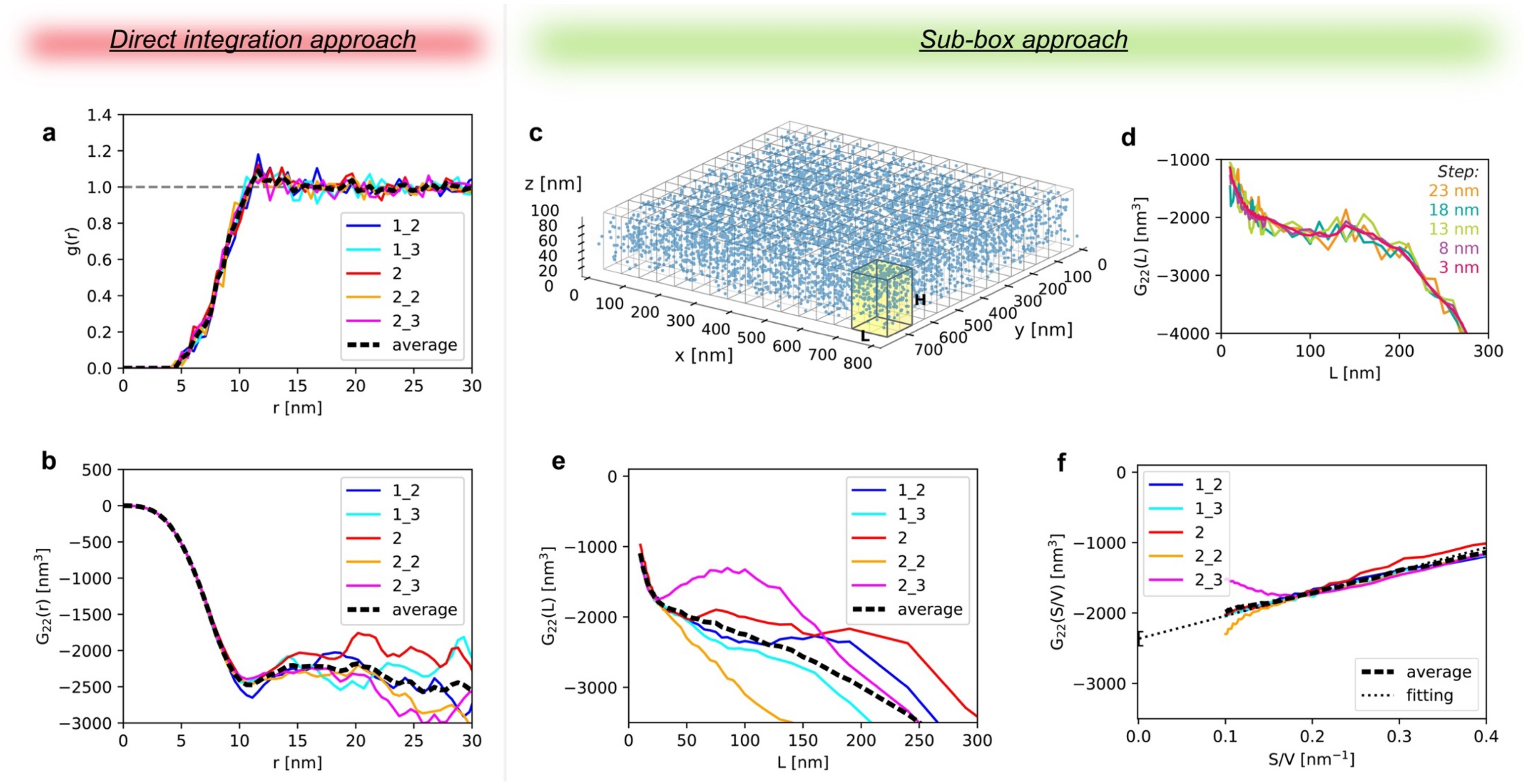

Figure SI6. Extraction of the KBI from BSA tomograms (16 mg/mL). **(a–b)** RDF and KBI (via direct integration of the RDF) as functions of centroid-to-centroid distance. **(c)** Example of the sub-box method applied to a BSA tomogram (L = 100 nm; step = 50 nm). **(d)** Influence of step on the effective KBI vs. box size. **(e)** Effective KBI vs. box size. **(f)** Effective KBI vs. sub-box surface-area-to-volume ratio (S/V). Legends in **(a, b, e, f)** denote tomogram labels.

9. Determination of the B22 by fitting the concentration dependence of $S(q = 0)$

Additionally, B_{22} can be found from the concentration dependence of the structure factor easily calculated from the cryo-ET tomograms. The fitting of $S(q)$ curves at low q by the second-order polynomials leads to finding the intercept of the fitting curve $S(q = 0)$. The B_{22} and KBI can be determined from the slope of the linear dependence of the reversed $S(q = 0)$ on particle number density ρ (**Figure SI7**) [51]:

$$\frac{1}{S(q=0,\rho)} = 1 + 2B_{22}\rho + (\tilde{o}) = 1 - G_{22,0}^{\infty}\rho + (\tilde{o}). \quad (8)$$

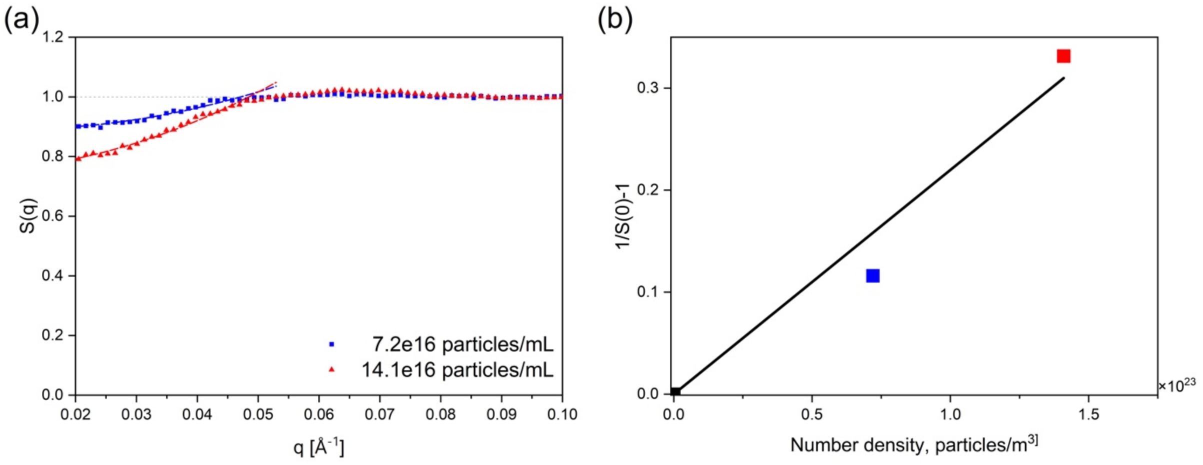

Figure SI7. (a) S(q) curves calculated from cryo-ET positions and fitted with a second-order polynomial for BSA. **(b)** The obtained intercept S_0values were reversed, decreased by 1, and plotted against protein number density. The slope of the linear fit corresponds to twice the second virial coefficient.

10. Results of the zeta potential measurements

Table SI5. Zeta potential values of BSA samples measured at 20 °C.

Sample	Zeta potential, mV	PIP
**1:** BSA in 5mM sodium phosphate buffer + 10 mM NaCl, pH 7.2	–9.3±0.8	Fig. 3a,b
**2:** BSA in 5mM sodium acetate buffer + 10 mM NaCl, pH 4.8	+0.2±4.1	Fig. 3b,d
**3:** sample **2** + 0.25 M proline, pH 4.8	+0.3±0.8	Fig. 3d
**4:** sample **2** + 0.5 M proline, pH 4.8	+0.1±1.0	Fig. 3d
**5:** sample **2** + 1 M proline, pH 4.8	–0.9±0.8	Fig. 3d

11. Tomogram of lysozyme and its segmentation

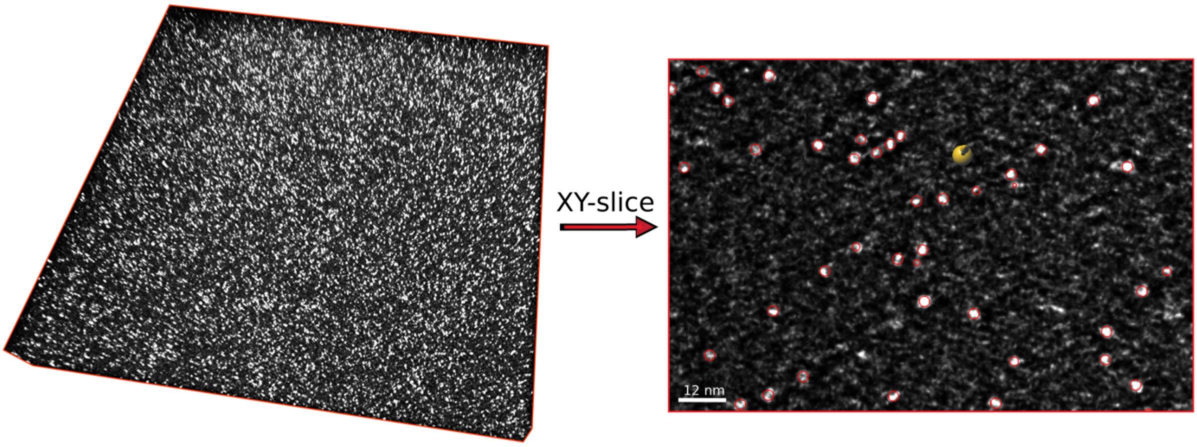

Figure SI8. Representative tomogram of lysozyme with a 7 nm–thick XY-slice; red circles indicate segmented protein particles.

12. Fitting of BSA U(r) using DLVO equation

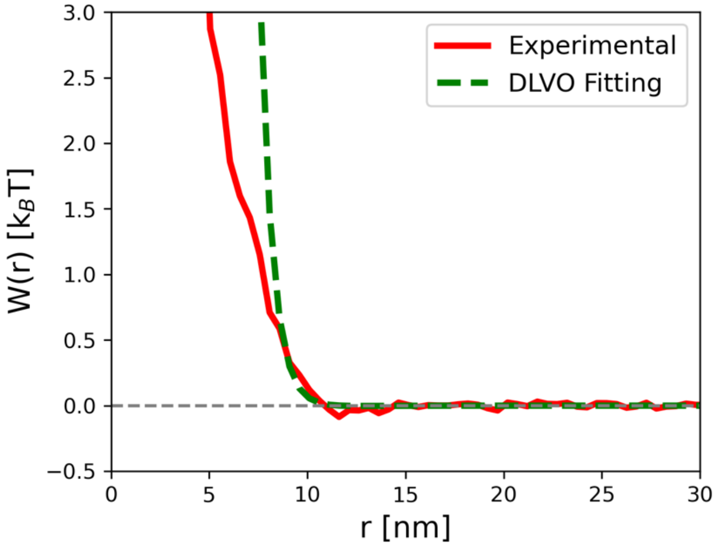

Figure SI9. Experimental protein interaction potential (PIP) of BSA measured by cryo-ET compared with the fit obtained using the DLVO equations for spherical particles **(eq. 1–4, below)**. Fitting parameters are given in **Table SI6**.

$$U_{el}(r) = \frac{(Ze)^2 \exp\left(-\frac{r-2R}{\lambda_D}\right)}{4\pi\varepsilon_0\varepsilon\left(1+\frac{R}{\lambda_D}\right)^2 r}, \tag{1}$$

$$\lambda_D = \sqrt{\frac{\varepsilon_0\varepsilon k_B T}{2e^2 N_A I}}, \tag{2}$$

$$U_{vdW}(r) = -\frac{A_H}{12}\left(\frac{4R^2}{r^2-4R^2} + \frac{4R^2}{r^2} + 2\ln\left[1 - \frac{4R^2}{r^2}\right]\right), \tag{3}$$

$$U_{DLVO}(r) = U_{vdW}(r) + U_{el}(r), \tag{4}$$

where $U_{el}(r)$, $U_{vdW}(r)$, $U_{DLVO}(r)$ are the electrostatic, van der Waals, and total DLVO interaction potentials, respectively; r is the intercentroid distance; Z is the protein net surface charge; e is the elementary charge; R is the effective sphere radius; ε_0 is the vacuum permittivity; ε is the dielectric constant; λ_D is the Debye–Hückel length; I is the ionic strength; k_B is the Boltzmann constant; T is the temperature; N_A is Avogadro's number; and A_H is the Hamaker constant.

Table SI6. Results of fitting the experimental PIP curves for BSA using the DLVO equation.

Parameter	Fitted value
A_H $[k_BT]$	8.42
R [nm]	3
Z [elementary charges]	92.6
ε	77

13. Summary on proteins and sample conditions

Table SI7. Physicochemical properties of proteins used in this work.

Protein	MW [Da]	Dimensions [nm × nm × nm]	Hydrodynamic diameter [nm]	Isoelectric point	Concentrations in this work [mg/mL]
BSA	66,500	7.5 × 6.5 × 4.0[a]	7.1[a]<br>6.8[e]<br>6.7[e]<br>6.6–8.6[f]	4.7–4.9[j]	8<br>16
Lys	14,300	3.0 × 3.0 × 4.5[b]	4[e]<br>3.8[g]	10.8–11.4[k]	4<br>6
OVA	42,700	7.0 × 3.6 × 3.0[a]	6.1[a]<br>6[e]<br>5.7[e]	4.5–4.7[l,m]	6<br>15<br>25
SAv	55,000	5.8 × 5.4 × 4.8[c]	5[h]	6.1–7.5[n]	10<br>15
Hb	64,500	6.0 × 5.0 × 5.0[a]	6.6[e]	6.9–7.4[j]	17<br>36
HSA	66,400	7.5 × 6.5 × 4.0[a]	7[a]	4.6–5.3[j]	6<br>22<br>31
ApoF	440,000	12[d]	12.2[e]<br>12.7[i]	4.0[i]	32<br>50

[a]Erickson, H. P. *Biol. Proced. Online* **2009**, 11, 32.
[b]Taylor, D. J. F.; Thomas, R. K.; Penfold, J. *Adv. Colloid Interface Sci.* **2007**, 132, 69.
[c]Ren, C.-L.; Carvajal, D.; Shull, K. R.; Szleifer, I. *Langmuir* **2009**, 25 (20), 12283–12292.
[d]Kim, M.; Rho, Y.; Jin, K. S.; Ahn, B.; Jung, S.; Kim, H.; Ree, M. *Biomacromolecules* **2011**, 12 (5), 1629–1640.
[e]Uversky, V. N. *Biochemistry* **1993**, 32 (48), 13288–13298.
[f]Jachimska, B.; Wasilewska, M.; Adamczyk, Z. *Langmuir* **2008**, 24 (13), 6866–6872.
[g]Parmar, A. S.; Muschol, M. *Biophys. J.* **2009**, 97, 590.
[h]Chirra, H. D.; et al. *Acta Biomater.* **2011**, 7, 2865.
[i]Petsev, D. N.; Thomas, B. R.; Yau, S.-T.; Vekilov, P. G. *Biophys. J.* **2000**, 78 (4), 2060–2069.
[j]Malamud, D.; Drysdale, J. W. *Anal. Biochem.* **1978**, 86 (2), 620–647.
[k]Koehler, J. A.; Ulbricht, M.; Belfort, G. *Langmuir* **1997**, 13 (15), 4162–4171.
[l]Li, Z. K.; Kuang, H.; Yang, J.; Hu, J.; Ding, B.; Sun, W.; Luo, Y. *Sci. Rep.* **2020**, 10 (1), 3456.
[m]Paul, B.; Furst, E. M.; Lenhoff, A. M.; Gilbert, E. P.; Wagner, N. J.; Teixeira, S. C. M. *J. Food Process Eng.* **2025**, 48 (4), e70088.
[n]Jeon, B. J.; Kim, S.; Kim, M.-S.; Lee, J.-H.; Kim, B. S.; Hwang, K. Y. *IUCrJ* **2021**, 8 (2), 168–177.

Table SI8. Conditions used to prepare samples in this work.

Conditions					Fig. in the main text
**Buffer**	**Extra component**	**pH**	**Ionic strength [mM]**	**Temperature [°C]**	
76 mM PB[a]	–	7.2	175	20	2, 3c, 4a-e
5 mM PB[b]	10 mM NaCl	7.2	21	20	3a,b
5 mM PB	975 mM NaCl	7.2	986	20	3a
5 mM AcB[c]	10 mM NaCl	4.8	15	20	3b,d
76 mM PB	–	7.2	175	4	3c
76 mM PB	–	7.2	175	40	3c
5 mM AcB	10 mM NaCl<br>0.25 M proline	4.8	15	20	3d
5 mM AcB	10 mM NaCl<br>0.5 M proline	4.8	15	20	3d
5 mM AcB	10 mM NaCl<br>1 M proline	4.8	15	20	3d
10 mM AcB	–	5	10	20	4f

[a]Phosphate buffer (**PB**) which contains 49.5 mM of Na_2HPO_4 and 26.3 mM NaH_2PO_4.
[b]Phosphate buffer which contains 3.1 mM of Na_2HPO_4 and 1.9 mM NaH_2PO_4.
[c]Acetate buffer (**AcB**)

14. Effect of the tilt-series parameters on the PMF

Object. We used gold nanoparticles (AuNPs) as a high-contrast standard that enables reliable particle detection and accurate centroid localization.

Procedure. Starting from a tilt series acquired with a −60°/2°/+60° scheme, we aligned the full dataset and reconstructed additional tomograms after excluding subsets of tilts to emulate ±40°/2°, ±20°/2°, and ±40°/4° schemes. For each reconstruction, AuNPs were segmented in Imaris using Surface detection with the "split spots" option. We then computed the PMF for each case. In addition, to compare localization fidelity independent of detection yield, we selected subtomograms in which the same number of particles was detected across all schemes and compared the resulting pair-distance distributions.

Tilt-range impact. While, in principle, centroid coordinates can be recovered even from very few projections,[1] reducing the maximum tilt angle increases missing-wedge effects and associated anisotropy/distortions. Consistent with this, pair-distance distributions for ±40° and ±60° were essentially identical (**Fig. SI10a**), whereas at ±20° the distribution broadened (**Fig. SI10b**). The most detrimental consequence at low maximum tilt (±20°) was that closely spaced particles frequently merged into a single segmented object (**Fig. SI10c**). Accordingly, PMFs matched well between ±40° and ±60° but deviated for ±20° (**Fig. SI10d**). Overall, ±60° would be preferable; however, in our setup the Chameleon grid geometry (copper wires) limits the achievable tilt range. Importantly, ±40° is beyond sufficient to recover correct pair distances and PMFs in our system.

Tilt-increment impact. Comparing 2° and 4° increments (±40°/2° vs ±40°/4°) yielded similar PMFs overall, but the 4° increment resulted in a loss of positional information, consistent with less accurate particle coordinates (**Fig. SI10e,f**).

(1) Jagota, M.; Townshend, R. J. L.; Kang, L.-W.; Bushnell, D. A.; Dror, R. O.; Kornberg, R. D.; Azubel, M. Gold Nanoparticles and Tilt Pairs to Assess Protein Flexibility by Cryo-Electron Microscopy. Ultramicroscopy 2021, 227, 113302. https://doi.org/10.1016/j.ultramic.2021.113302.

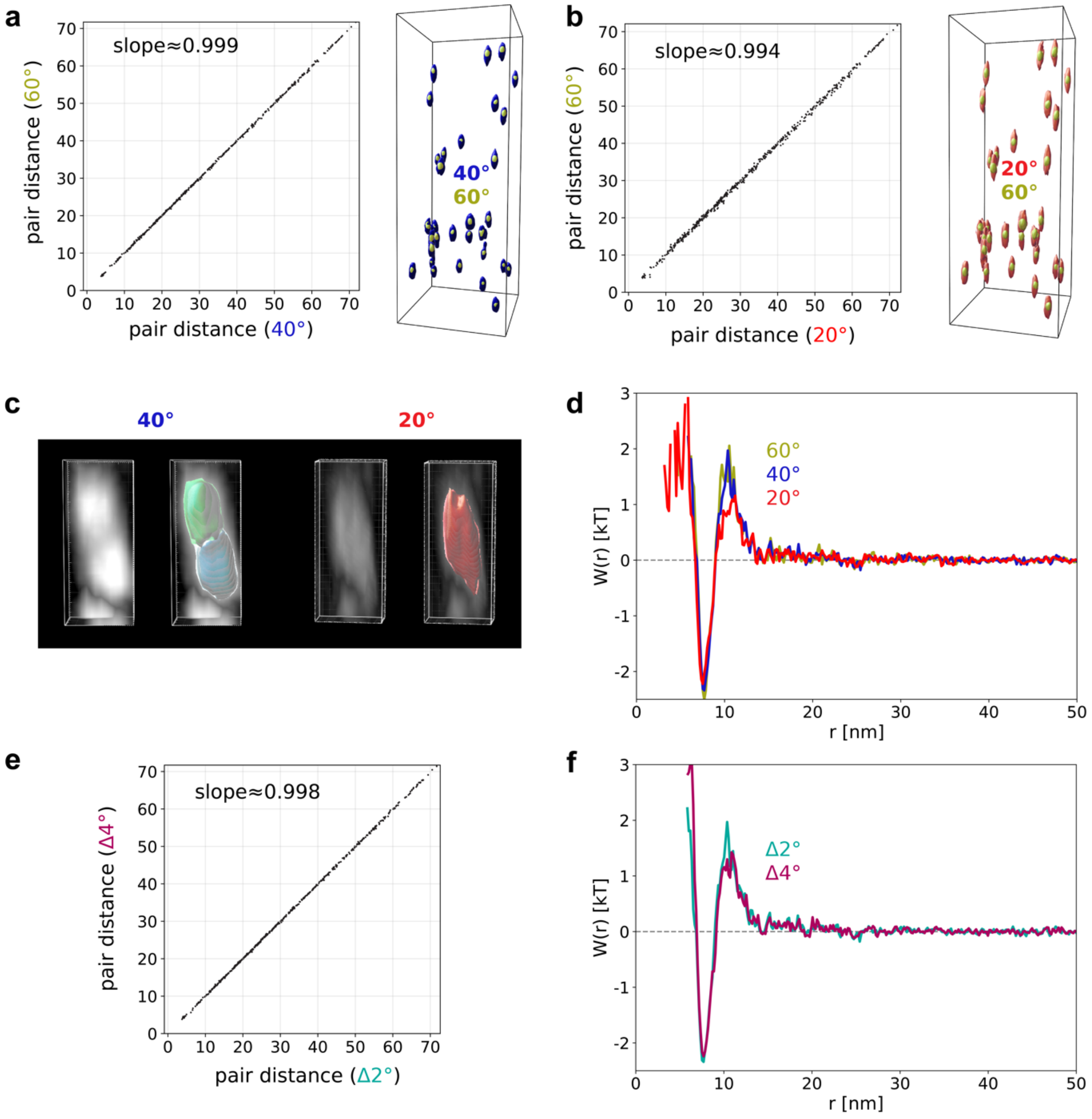

Figure SI10. Effect of the tilt-series parameters on the PMF of gold nanoparticles: (a) pair-distance correlation between maximum tilt angles of 40° and 60°; (b) pair-distance correlation between maximum tilt angles of 20° and 60°; (c) example of a problematic reconstruction of the same particle from tilt series with maximum tilt angles of 40° and 20°; (d) PMF as a function of centroid-to-centroid distance for three maximum tilt angles (20°, 40°, 60°); (e) pair-distance correlation for increment angles of 2° and 4°; (f) PMF as a function of centroid-to-centroid distance for increment angles of 2° and 4°.

15. Segmentation of apoferritin

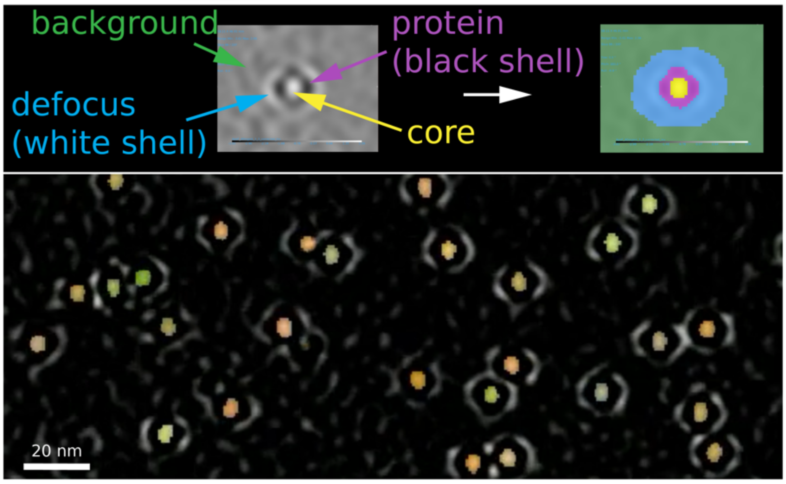

Figure SI11. (**Top**) Schematic representation of the classes used to train the neural network for determining the centroid position of apoferritin. (**Bottom**) Example of successful segmentation on a fragment of an XY-slice of the apoferritin tomogram.
